# The transcriptional regulator CtrA controls gene expression in Alphaproteobacteria phages: Evidence for a lytic deferment pathway

**DOI:** 10.3389/fmicb.2022.918015

**Published:** 2022-08-19

**Authors:** Elia Mascolo, Satish Adhikari, Steven M. Caruso, Tagide deCarvalho, Anna Folch Salvador, Joan Serra-Sagristà, Ry Young, Ivan Erill, Patrick D. Curtis

**Affiliations:** ^1^Department of Biological Sciences, University of Maryland Baltimore County, Baltimore, MD, United States; ^2^Department of Biology, University of Mississippi, Oxford, MS, United States; ^3^Keith R. Porter Imaging Facility, College of Natural and Mathematical Sciences, University of Maryland Baltimore County (UMBC), Baltimore, MD, United States; ^4^Universitat Autònoma de Barcelona, Cerdanyola del Vallès, Barcelona, Spain; ^5^Center for Phage Technology, Texas A&M University, College Station, TX, United States

**Keywords:** bacteriophage, lysis, pseudolysogeny, cell cycle, regulation, transcription, CtrA, Caulobacter

## Abstract

Pilitropic and flagellotropic phages adsorb to bacterial pili and flagella. These phages have long been used to investigate multiple aspects of bacterial physiology, such as the cell cycle control in the Caulobacterales. Targeting cellular appendages for adsorption effectively constrains the population of infectable hosts, suggesting that phages may have developed strategies to maximize their infective yield. Brevundimonas phage vB_BsubS-Delta is a recently characterized pilitropic phage infecting the Alphaproteobacterium *Brevundimonas subvibrioides*. Like other Caulobacterales, *B. subvibrioides* divides asymmetrically and its cell cycle is governed by multiple transcriptional regulators, including the master regulator CtrA. Genomic characterization of phage vB_BsubS-Delta identified the presence of a large intergenic region with an unusually high density of putative CtrA-binding sites. A systematic analysis of the positional distribution of predicted CtrA-binding sites in complete phage genomes reveals that the highly skewed distribution of CtrA-binding sites observed in vB_BsubS-Delta is an unequivocal genomic signature that extends to other pilli- and flagellotropic phages infecting the Alphaproteobacteria. Moreover, putative CtrA-binding sites in these phage genomes localize preferentially to promoter regions and have higher scores than those detected in other phage genomes. Phylogenetic and comparative genomics analyses show that this genomic signature has evolved independently in several phage lineages, suggesting that it provides an adaptive advantage to pili/flagellotropic phages infecting the Alphaproteobacteria. Experimental results demonstrate that CtrA binds to predicted CtrA-binding sites in promoter regions and that it regulates transcription of phage genes in unrelated Alphaproteobacteria-infecting phages. We propose that this focused distribution of CtrA-binding sites reflects a fundamental new aspect of phage infection, which we term lytic deferment. Under this novel paradigm, pili- and flagellotropic phages exploit the CtrA transduction pathway to monitor the host cell cycle state and synchronize lysis with the presence of infectable cells.

## Introduction

Following infection, virulent phages replicate and lyse the cell to release virions. Temperate phages, on the other hand, have the additional option of lysogeny, in which phage gene expression is repressed and a prophage is established *via* integration into the bacterial chromosome or as a plasmid (Howard-Varona et al., 2017). The phage infection process starts with the adsorption of phage particles to the bacterial cell. Adsorption is mediated through interactions between phage binding proteins and bacterial cell surface receptors, which are typically components of the bacterial cell wall (Bertozzi Silva et al., 2016). Some phages, however, initiate adsorption through interactions with bacterial pili or flagella (Bradley, 1972; Lagenaur et al., 1977; Frost, 1993). Pili-and flagellotropic phages include several major bacteriophage families and pilitropic phages are known to target both plasmid- and chromosomally encoded pili, suggesting that pilus-mediated adsorption has evolved independently in several phage lineages (Ceyssens and Lavigne, 2010).

Pilitropic phages played an important role in the investigation of the cell cycle of the Alphaproteobacterium *C. crescentus*. This bacterium has a dimorphic lifestyle, producing two different cell types after division: a surface-attached sessile “stalked” cell and a motile “swarmer” cell with a polar flagellum surrounded by polar pili. Bacteriophage ΦCbk and related pilitropic phages target the polar flagellum and pili of swarmer cells, and ΦCbk was instrumental in unraveling the genetic regulation of pili production (Skerker and Shapiro, 2000).

Cell-cycle control in *C. crescentus* is orchestrated by a complex network of transcriptional, epigenetic and proteolytic interactions involving several global regulators; including CtrA, DnaA, GcrA, CcrM, CckA and DivK (Brilli et al., 2010). Among these, CtrA, CcrM, DnaA and GcrA are widely conserved across the Alphaproteobacteria that have not undergone massive genomic reduction. CtrA is the master regulator of development in *C. crescentus*, having critical roles in the regulation of cell division, chromosomal replication, pilus assembly and flagellar biosynthesis (Laub et al., 2002). CtrA regulates transcription of target genes by binding a conserved palindromic motif with consensus sequence TTAA-N7-TTAA (Ouimet and Marczynski, 2000). The conservation of this transcriptional regulator and its motif in multiple Alphaproteobacteria with simpler morphogenetic life cycles suggested that its primary function might not be cell-cycle regulation (Brilli et al., 2010). Subsequent work has shown that the ancestral regulatory role of CtrA is likely the control of motility and chemotaxis (Bird and MacKrell, 2011; Greene et al., 2012; Zan et al., 2013, 1; Wang et al., 2014; Francez-Charlot et al., 2015; Hernández-Valle et al., 2020).

Phages use multiple strategies to exert regulatory control on the transcription of many bacterial genes (Yang et al., 2014). In contrast, host transcriptional regulation of phage genes has not been so amply documented and is typically associated with phage adaptation to cellular stress. A canonical example of this phenomenon is the activation of the lytic cycle in temperate phages in response to DNA damage. Phage transcriptional regulators, epitomized by the Enterobacteria phage lambda CI repressor, regulate the activity of lytic phase genes by competing for promoter occupancy with Cro-type repressors (Ptashne, 1992). The presence of DNA damage, as a result of different stresses, results in the autocatalytic cleavage of CI-like repressors, leading to the onset of the phage lytic cycle (Sauer et al., 1982). Recent work has shown that phages can incorporate additional layers of logic to this decision making, using quorum sensing-like mechanisms to reduce prophage induction at high phage concentrations (Brady et al., 2021).

Several phages have independently evolved the ability to exploit a bacterial transcriptional regulator, the LexA repressor, to trigger their lytic cycle (Fornelos et al., 2016). Like CI, LexA undergoes RecA-mediated autocatalytic cleavage in the presence of DNA damage, de-repressing its target genes and inducing the bacterial SOS response (Erill et al., 2007). Induction of the lytic cycle in these phages is mediated by the presence of LexA-binding sites in the promoter region of antirepressor, co-repressor or lytic phase genes in the phage chromosome (Fornelos et al., 2011). Co-option of host transcription factors to regulate phage gene expression is not restricted to activation of the phage lytic cycle by LexA. The virulent Bacillus phage Φ29 contains multiple promoter-associated binding sites for the master regulator of sporulation initiation Spo0A. Binding of Spo0A to these sites suspends lytic cycle development, enabling spore entrapment of the Φ29 chromosome by the chromosome segregation protein Spo0J (Meijer et al., 2005). The phage can thus become dormant during nutrient starvation and resume its lytic cycle following spore germination. These two cases illustrate the ability of phages to exploit host-encoded signal transduction systems to control phage gene expression and modulate its lifestyle in response to cellular states.

Here we report on the genomic characterization of a novel phage infecting the Alphaproteobacterium *Brevundimonas subvibrioides* that harbors a large intergenic region encompassing multiple putative CtrA-binding sites. Comparative genomics analyses reveal that most pilitropic phages infecting Alphaproteobacteria species contain genomic regions enriched in putative CtrA-binding sites, which are preferentially located in promoter regions. Our results show that CtrA binds to these predicted sites to regulate the expression of genes in several unrelated phages that infect different Alphaproteobacteria genera, suggesting that CtrA regulation of phage genes has evolved independently and hence likely conveys a selective advantage to pilitropic phages.

## Materials and methods

### Genome data collection

Complete genome sequences for all bacteriophages analyzed in this work were retrieved from the NCBI GenBank (GenBank, RRID:SCR_002760) and RefSeq (RefSeq, RRID:SCR_003496) databases (Benson et al., 2017; Haft et al., 2018), prioritizing RefSeq records for a given bacteriophage whenever both were available. When annotated, the 5′ instance of Direct Terminal Repeat (DTR) regions was removed from the resulting genome objects to avoid double counting. Bacteriophage genomes potentially related to Brevundimonas phage vB_BsubS-Delta were identified by serially querying the NCBI GenBank database with tBLASTN (NCBI BLAST, RRID:SCR_004870), restricted to taxonomy ID 10239 (Viruses), using each of the annotated vB_BsubS-Delta proteins as the query sequence (Altschul et al., 1997). Only tBLASTN hits with e-value lower than 10^−10^ and query coverage larger than 0.75 were considered, and the nucleotide sequence of the genome encoding the putative homologous protein was retrieved from NCBI GenBank/RefSeq. Identifiers for the genome sequences of bacteriophages infecting Alphaproteobacteria hosts were obtained from INPHARED (Jan 2021; Cook et al., 2021). Identifiers for the genome sequences of pilitropic bacteriophages were obtained from a targeted search on the NCBI PubMed service (PubMed, RRID:SCR_004846), followed by manual curation of reference articles. Genome sequences were subsequently downloaded from the NCBI GenBank/RefSeq databases. Only sequences at least 10,000 bp-long were used for analysis.

### Genomic sequence analyses

Phage lifestyle (temperate vs. virulent) was predicted using the BACterioPHage LIfestyle Predictor (BACPHLIP) classifier (Hockenberry and Wilke, 2021) with default parameters. Multiple sequence alignment of DTR regions was performed using progressive Mauve (Darling et al., 2004), following the extraction of DTRs from genome sequences using their GenBank annotation. Pairwise nucleotide sequence identity values for DTRs were computed using the ViPhy pipeline to identify homologous sequence segments, using a limiting BLAST e-value threshold of 10, and normalizing identity counts on homologous segments to the overall sequence length.

### Identification of putative transcription factor-binding sites

A collection of experimentally validated CtrA-binding sites was downloaded from the CollecTF database (CollecTF, RRID:SCR_014405; Kılıç et al., 2014). Binding sites for GcrA, SciP and DnaA were obtained from previously published work (Hottes et al., 2005; Tan et al., 2010; Gonzalez et al., 2014; Haakonsen et al., 2015). Collections of TF-binding sites were then used to generate position-specific scoring matrix (PSSM) models to scan complete bacteriophage genomes with a custom Python script,^1^ as described previously (Hobbs et al., 2016). Only positions with a score above the Patser threshold, satisfying the equality between the negative logarithm of the false positive rate and the information content of the TF-binding motif, were considered as putative TF-binding sites (Hertz and Stormo, 1999).

### Computation of normalized positional entropy and Gini coefficient

To analyze the asymmetry in TF-binding site distribution along the genome, bacteriophage genomes were divided into 50 equal-sized bins and the number of putative TF-binding sites identified in each bin tallied and normalized to obtain site frequencies per bin. The normalized entropy of the distribution S of TF-binding sites along the genome was computed as follows:


(1) $$\begin{array}{l} \hat{\text{H}}\left(\text{S}\right)=\frac{\text{H}\left(\text{S}\right)}{\max_{\text{n}}\left(\text{H}\left(\text{S}\right)\right)}\;\\ \text{H}\left(\text{S}\right)=-\sum_{\text{i}=1}^{\text{b}}\text{f}\left({\text{s}}_{\text{i}}\right)\cdot \log_{2}\left(\text{f}\left({\text{s}}_{\text{i}}\right)\right) \end{array}$$

where f(s_i_) is the relative frequency of putative TF-binding sites in bin i, b is the number of bins, max_n_(H(S)) is the largest possible entropy, assuming the most even distribution for the number of sites observed in the genome, and Ĥ(S) is the resulting normalized entropy. This normalization addresses small number effects arising from low TF-binding site counts, which impact the maximum entropy attainable. The Gini coefficient (O’Neill and Erill, 2016) of the distribution S of TF-binding sites along the genome was computed as:


(2) $$\begin{array}{l} \hat{G}\left(S\right)=\frac{G\left(S\right)-\min_{n}\left(G\left(S\right)\right)}{\max_{n}\left(G\left(S\right)\right)-\min_{n}\left(G\left(S\right)\right)}\\ G\left(S\right)\;=\frac{\sum_{i=1}^{b}\sum_{j=1}^{b}\left|s_{j}-s_{i}\right|}{2\cdot b^{2}\langle s_{i}\rangle } \end{array}$$

where s_i_ is the number of sites in bin i, b is the number of bins and max(G(S)) and min(G(S)) are, respectively, the maximum and minimum Gini coefficients attainable given the number of TF-binding sites detected in the genome. To avoid binning artifacts, bins were defined as starting both at genome position 1 and at 1 + L/2, where L is the length of the bin for a given genome, with rollover. The maximum (entropy) or minimum (Gini) value obtained for the genome was then reported. Only phage genomes with more than 5 putative TF-binding sites were considered for analysis.

### Phylogenetic analysis

For phylogenetic analysis, bacteriophage genomes potentially related to bacteriophage vB_BsubS-Delta were complemented with the genome sequences of known Alphaproteobacteria bacteriophages and reported pilitropic and flagellotropic bacteriophages. A genome-based phylogeny was generated with the ViPhy Python pipeline.^2^ Agglomerative clustering was performed with scikit-learn (scikit-learn, RRID:SCR_002577) on the resulting tree using 50 target clusters (Pedregosa et al., 2011). Representative phage genomes for each cluster were selected and complemented with genomes of phages presenting skewed CtrA-binding site distribution. The final set of 100 phage genomes was used to generate a genome-based phylogeny with the VICTOR web service (Meier-Kolthoff and Göker, 2017). Inter-genomic protein sequence distances were computed with 100 pseudo-bootstrap replicates using the Genome-BLAST Distance Phylogeny (GBDP) method optimized (distance formula d_6_) for prokaryotic viruses (Meier-Kolthoff et al., 2013), and a minimum evolution tree was computed with FASTME on the resulting inter-genomic distances (Lefort et al., 2015). The resulting phylogenetic tree was annotated using different types of information (lifestyle, asymmetric distribution of CtrA-binding sites, known pili/flagellotropic nature) using the iTOL web service (iTOL, RRID:SCR_018174; Letunic and Bork, 2019).

### Statistics and data visualization

Differences in the distributions of putative TF-binding site scores and intergenic localization for selected datasets were analyzed using a one-sided Mann–Whitney U test. Positional asymmetry maps and distribution violin plots were generated with Python scripts.^3^ Comparative genome plots were generated using the BLAST Ring Image Generator (BRIG, RRID:SCR_007802; Alikhan et al., 2011). Genome maps were generated with DNAmaster (Pope and Jacobs-Sera, 2018).

### Electron microscopy

Phage lysate (10 μl) was deposited onto a 200 mesh formvar and carbon-coated copper grids (EMS). The lysate was allowed to set for 1 min, briefly rinsed with ultra-pure water and stained with 2% uranyl acetate for 2 min. Imaging was performed with a Morgagni 268 Transmission Electron Microscope (FEI, Hillsboro, IL, United States) equipped with an Orius CCD camera (Gatan Inc., Pleasanton, CA, United States). Head and tail measurements were performed with the Fiji software package (Fiji, RRID:SCR_002285; Schindelin et al., 2012).

### Extraction of phage genomic DNA and nuclease assays

Phage genomic DNA was purified following (Summer, 2009), using the Wizard DNA Clean-up Kit (Promega, RRID:SCR_006724). For general nuclease digestion, RNase-free DNase I and DNase-free RNase A were obtained from Thermo Scientific. For each digestion, 1 μg phage genomic DNA was used in combination with the DNase I buffer provided by the manufacturer. In the DNase reaction 1 U of enzyme was used, while the RNase reaction used 10 μg of that enzyme. Digestions were incubated at 37°C for 2 h, followed by gel electrophoresis. Double restriction digestion of phage genomic DNA with enzymes AflII and PciI was carried out according to manufacturer’s protocols (New England Biolabs, RRID:SCR_013517), followed by gel electrophoresis.

### Growth conditions and strains

All strains, plasmids and primers used in this study are listed in Supplementary Table S1. All *C. crescentus* strains were grown in PYE medium or plates (2 g/l peptone, 1 g/l yeast extract, 0.3 g/l MgSO_4_, 7H_2_O, 0.0735 g/l CaCl_2_, 2H_2_O, 1.5% agar for plates) at 30°C or 37°C. Antibiotics were added when necessary. Tetracycline was added at 1 μg/ml. *Escherichia coli* strains were grown in LB media (10 g/l tryptone, 5 g/l yeast extract, 10 g/l NaCl, 1.5% agar for plates) at 37°C. Tetracycline was added at 12 μg/l when required.

### CtrA purification and electrophoretic mobility shift assay

For CtrA purification, the coding region of *ctrA* was amplified from *C. crescentus* using primers ctrApET28aF and ctrApET28aR. The amplified region was cloned into pET28a plasmid using Gibson Assembly to create a 6X his tag on the N terminus of CtrA (pAR0001). This plasmid was introduced into *E. coli* BL21 (DE3) strain by electroporation. CtrA purification was performed by following the QIAexpressionist manual (QIAGEN, RRID:SCR_008539) under native conditions. DNA probes based on phage sequences were synthesized (Supplementary Table S1), with the forward strand receiving 5′ biotinylation. Forward and reverse strands were mixed, heated and gently cooled to create double-stranded probes. For DELTA_120, a probe was designed that lacked biotinylation. For other phage DNA probes, probes were designed where the CtrA binding sites were mutated such that the TTAA-N7-TTAA CtrA-binding sites were replaced with GGCC-N7-GGCC.

To perform the EMSA, 7.5 μl CtrA at a final concentration of 3.3 μm was phosphorylated by incubation with 1 μl ATP (1 mm final concentration) and 1 μl of 7.5 μm MBP-EnvZ (final concentration 0.75 μm) in a buffer consisting of 50 mm Tris HCl (pH 7.8), 50 mm KCl, 20 mm MgCl_2_, 1 mm DTT, at 30°C for 20 min. This is a modified protocol of (Reisenauer et al., 1999). Double stranded biotin-labeled DNA (50 ng) was then mixed with 2 μl Binding buffer [50 mm KCl, 5 mm MgCl_2_, 20 mm Tris–HCl (pH 8), 100 μm EDTA, 1 mm DTT, 10% glycerol] and 1 μg Poly dI-dC. Phosphorylated CtrA (5 μl) was then added to the mixture (final concentration 1.65 μm) making the total volume to 10 μl and it was incubated for 25 min at RT. Loading buffer [2 μl, 100 mm Tris–HCl (pH 7.8), 0.2% bromophenol blue] was then added to the mix just before loading. A 5% native PAGE gel was prepared with the following composition: 7.5 ml of 1X TBE (pH 8), 5.25 ml water, 2.25 ml 40% polyacrylamide, 6 μl TEMED, 150 μl of 10% APS. The gel was pre-run at 150 V for 5 min and then the samples were loaded. Electrophoresis was carried out at 300 V for 30 min in 1X TBE buffer (pH 8). The gel, blotting paper and positively charged nylon membrane were then incubated in 0.5X TBE buffer (pH 8.3) for 20 min in a shaking platform at 4°C. Transfer was carried out using semi-dry electrophoretic transfer cell (Biorad) at 25 V, 1A for 40 min. Post transfer, the DNA was crosslinked to the membrane by exposure to UV short wave (254 nm) for 20 min using a hand held UV device. Next, the membrane was incubated in blocking buffer (Tropix I-Block, Applied Biosystems) supplemented with 0.2% Tween-20 on a shaking platform for 1 h at RT. The membrane was then incubated in blocking buffer with 0.2% Tween-20 and 1.5 μl of 1.25 mg/ml HRP-conjugated streptavidin at a dilution of 1:23000, on a shaking platform for 1 h at RT. Five consecutive washes were then carried out, each for 5 min on a shaking platform at RT, with 1X TBST (0.2% Tween-20). The membrane was then incubated with 3 ml of Supersignal West Pico PLUS Chemiluminescent Substrate for 10 min prior to imaging.

### Strain construction and beta-galactosidase assays

The promoter region of Brevundimonas phage vB_BsubS-Delta DELTA_120 gene was amplified using primers delta120betagalF and delta120betagalR, and cloned into the plac290 plasmid (Gober and Shapiro, 1992) using Gibson Assembly to create plasmid pSA1002. The promoter region of genes CbK_gp005 and CbK_gp018 from Caulobacter phage ΦCbk was amplified using primer-pairs phicbkgp5promoterF and phicbkgp5promoterR, and cbkgp18plac290F and cbkgp18plac290R, respectively, and cloned into plac290 plasmid using Gibson Assembly to create plasmids pSA1003 and pSA1004. The promoter region of Cp1R7AA1_019 from Mesorhizobium phage Cp1R7A-A1 was amplified using primers Mesopromoter019F and Mesopromoter019R and cloned into plac290 plasmid using Gibson Assembly to create plasmid pSA1005. Plasmids were introduced into *C. crescentus* NA1000 (WT) and LS2195 (*ctrA* temperature sensitive) strains using electroporation.

For beta-galactosidase assays, cells were grown up to mid log stage. Z buffer (60 mm Na_2_HPO_4_.7H_2_O, 40 mm NaH_2_PO_4_.H2O, pH 7.0, 10 mm KCl, 1 mm MgSO_4_.7H_2_O, 50 mm β-mercaptoethanol) and 50 μl of chloroform was added to 200 μl of cells to a final volume of 850 μl and incubated at 30°C for 5 min. This was followed by addition of 200 μl ONPG (4 mg/ml) and the reaction was allowed to proceed until yellow coloration started to develop. This time duration for the appearance of yellow coloration was noted and the reaction was stopped by addition of 400 μl sodium carbonate (1 M). This was followed by centrifugation (12,000 × g, 5 min) at room temperature and the absorbance at 420 nm of the supernatant was measured. Miller units for each reaction were calculated using the Miller formula (A_420_ × 1,000)/[OD_600_ × time (min) × volume of cells used (ml)]. Reactions were performed in triplicate and average and standard deviation were calculated.

### Genome sequencing, assembly and annotation

Genome annotation was performed with DNA Master (v5.23.3), using default settings (Pope and Jacobs-Sera, 2018). Automated gene calls from DNA Master were manually reviewed and further validated with ARAGORN (v1.2.38; Aragorn, RRID:SCR_015974), tRNAscan-SE (v2.0; tRNAscan-SE, RRID:SCR_010835) and GeneMarkS (v3.25; GeneMark, RRID:SCR_011930; Besemer et al., 2001; Laslett and Canback, 2004; Chan and Lowe, 2019). Functional annotations were completed using the NCBI BLASTP (NCBI BLAST, RRID:SCR_004870) and HHPred (HHpred, RRID:SCR_010276) services with default parameters, following SEA-PHAGES annotation guidelines (maximum BLASTP e-value: 10–7, minimum HHpred probability: 90%) and independently curated by at least two annotators (Altschul et al., 1997; Söding et al., 2005; Pope et al., 2014).

## Results

### Morphology and genome architecture of Brevundimonas phage vB_BsubS-Delta

Brevundimonas phage vB_BsubS-Delta was isolated from a freshwater pond in Lafayette County, Mississippi, and shown to have a narrow host range, infecting only four Brevundimonas species and none of the Caulobacter species tested (Sperling et al., 2019). Host mutants defective in pilus-biogenesis were shown to be resistant to vB_BsubS-Delta, suggesting that this phage utilizes pili to infect the cell (Sperling et al., 2019). Here we used transmission electron microscopy (TEM) and next-generation sequencing to assess the morphology and genomic architecture of vB_BsubS-Delta.

**Figure 1:**
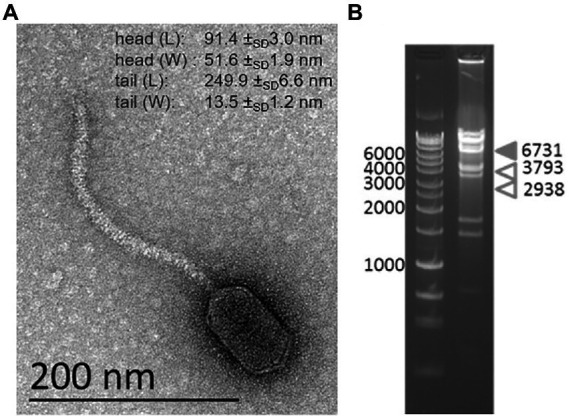
Characterization of Brevundimonas phage vB_BsubS-Delta. **(A)** TEM image of vB_BsubS-Delta from crude lysate. Scale bar = 200 nm. Average head and tail length/width measurements from four independent TEM images. **(B)** Restriction digestion of vB_BsubS-Delta genomic DNA with AflII and PciI to determine if the genome is linear or circular. Expected band sizes are indicated by arrows.

TEM imaging of vB_BsubS-Delta reveals a canonical siphophage morphology with long, non-contractile tails and an extremely prolate head (Figure 1A), reminiscent of the Caulobacter ΦCbk-like phages (Gill et al., 2012). Phage vB_BsubS-Delta has a head width of 51.6 ± _SD_1.9 nm, consistent with ΦCbk-like phages, but the head length is considerably shorter (91.4 ± _SD_3.0 nm) than that of ΦCbk-like phages (205–292 nm), suggesting a smaller genome size. Extracted genomic nucleic acid was subjected to digestion by either DNase I or RNase A. While DNase led to complete destruction of genomic material, RNase had no effect, suggesting that the vB_BsubS-Delta genome was DNA.

Genome sequencing resulted in an 87,225 bp-long linear genome assembly with distinct ends. To determine if the genome was circular, a restriction digest was carried out on the genome using enzymes AflII and PciI, which should define termini fragments of 2,938 and 3,793 bp in the assembled linear genome. Instead, two bands of approximately 4,000 bp were observed corresponding to the internal digestion fragments of 3,448 and 4,497 bp, as well as a band of approximately 6,731 bp corresponding to the cumulative size of the two termini fragments (Figure 1B). This strongly suggests that vB_BsubS-Delta has a circularly permuted genome, in contrast to ΦCbk-like phages, which have linear genomes with large direct terminal repeats (DTR; Gill et al., 2012).

**Figure 2:**
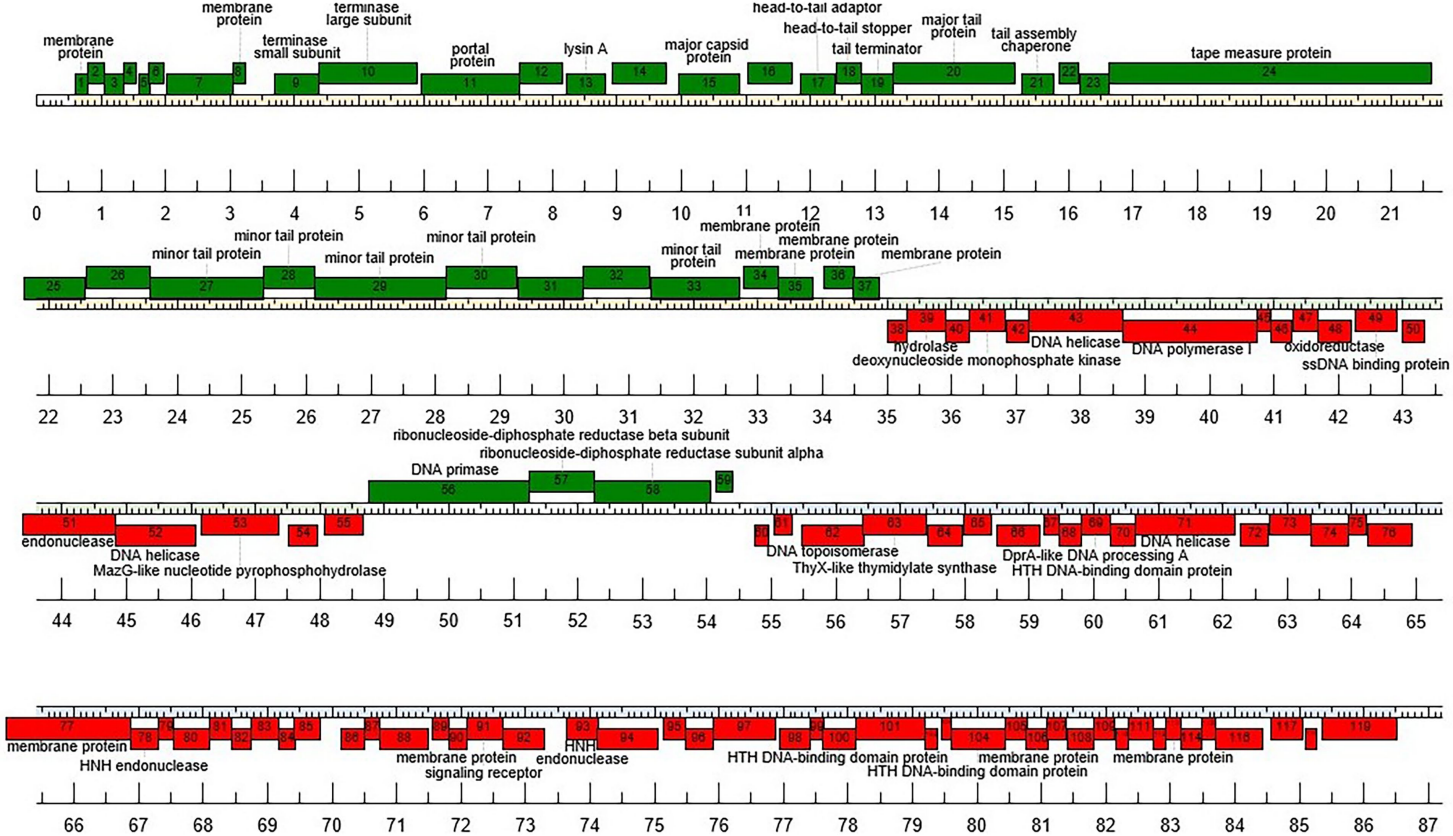
Genome organization of Brevundimonas phage vB_BsubS-Delta. Predicted genes are represented as boxes above or below the genome, denoting, respectively, rightwards- and leftwards-transcription.

Functional annotation of the vB_BsubS-Delta genome revealed a genome architecture distinct from that of ΦCbk-like phages (Figure 2). The vB_BsubS-Delta genome contains a well-defined phage structural protein module (DELTA_1-DELTA_37) encompassing tail and capsid components, the tape measure and portal proteins, and both terminase subunits located at the start of the module. The structural module also contains a Lysin A protein (DELTA_13). No independent lysis module was detected in the genome, but two of several predicted membrane proteins encoded at the end of the structural module (DELTA_34 and DELTA_35) present homology to, respectively, phage holins and spanins, suggesting that they may constitute a separate lysis module. The phage replication module (DELTA_38-DELTA_55) contains a Pol I-type DNA polymerase, as well as DNA helicases, a single-strand DNA-binding protein and a deoxynucleoside monophosphate kinase. Additional genes involved in DNA synthesis and replication (DNA primase) are located in a small divergently transcribed module (DELTA_56-DELTA_59) adjacent to the primary replication module.

A separate module (DELTA_60-DELTA_119) divergently adjacent to the terminase-end of the structural module includes several genes encoding proteins likely involved in packaging-related DNA processing, including two HNH endonucleases, DNA topoisomerase, DNA helicase and DNA processing protein A, as well as a ThyX-like thymidylate synthase homolog. A large (1,312 bp) intergenic region separates this ancillary packaging module from the divergently transcribed structural module.

### Phylogenetic analysis of vB_BsubS-Delta

Despite the morphological, receptor and host similarities between vB_BsubS-Delta and ΦCbk-like phages, the genomic organization of vB_BsubS-Delta, together with its smaller genome size, absence of tRNA genes and lower %GC content (62.49 %GC) suggested that this phage was not closely related to ΦCbk-like phages. To investigate the possible relatedness of vB_BsubS-Delta to ΦCbk-like and other phages, we performed whole-proteome phylogenetic inference. The analysis included 300 phage genomes partially related to vB_BsubS-Delta, identified *via* BLASTP with all vB_BsubS-Delta proteins as queries (Supplementary Table S2), representative complete sequences of 362 Alphaproteobacteria-infecting phages (Supplementary Table S3), and additional genomes for known pilitropic and flagellotropic phages (Supplementary Table S4). A consensus phylogenetic tree for the 460 phage genomes was generated with ViPhy (Supplementary Figure S1), and representative genomes for each cluster identified *via* agglomerative clustering. A final set of 100 phage genome sequences (Supplementary Table S5) was used to perform whole-genome phylogenetic inference using the VICTOR web service.

**Figure 3:**
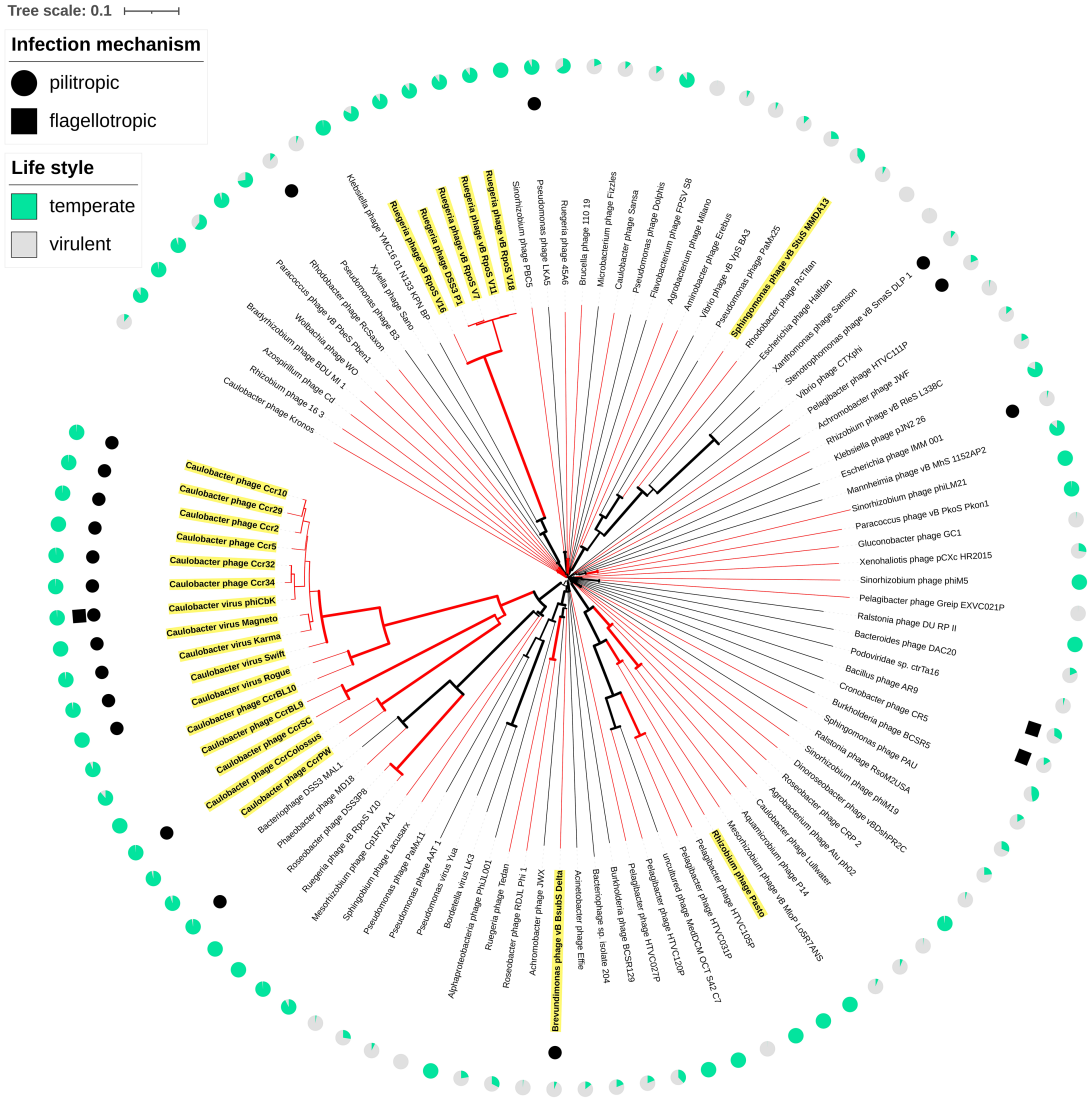
Whole-genome phylogeny of vB_BsubS-Delta. Whole-proteome tree of complete bacteriophage genomes. The tree was generated with FASTML using BLAST-derived inter-genomic distances using VICTOR. Midpoint rooting was applied for circular visualization. Bootstrap support values are shown as branch widths, with the thickest branches corresponding to 100% bootstrap support. The branches corresponding to Alphaproteobacteria-infecting phages are colored in red. External filled circles and squares designate, respectively, known pilitropic and flagellotropic phages (Supplementary Table S4). Names for LPEG phages (Supplementary Table S7) are highlighted in yellow. External pie-charts indicate (green) the probability of temperateness assigned by the BACPHLIP algorithm (Supplementary Table S10).

**Figure 4:**
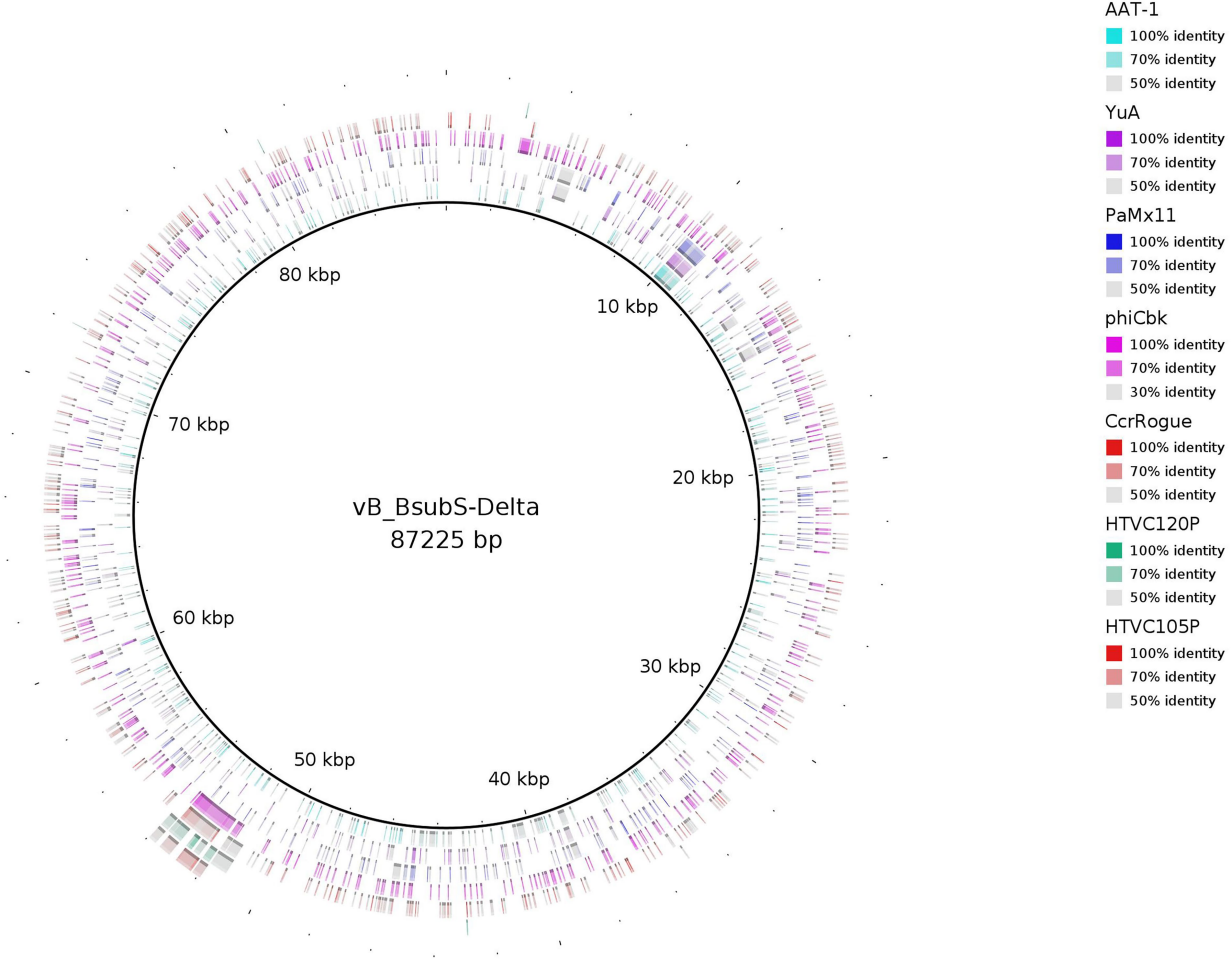
Comparative genome map of representatives from major phylogenetic clusters using Brevundimonas phage vB_BsubS-Delta as a reference. The percent amino acid identities of each phage relative to Brevundimonas phage vB_BsubS-Delta, as identified by tBLASTX analysis, are shown as different hues. Concentric rings correspond to phages listed in the legend, with the innermost ring corresponding to Pseudomonas phage AAT-1 and the outermost ring corresponding to Pelagibacter phage HTVC105P. Segments with percent identity below 50% are not shown.

The consensus phylogenetic tree shown in Figure 3 (Supplementary Figure S1) reveals that phage vB_BsubS-Delta is not directly related to any previously sequenced phages, placing it in a long branch within a well-supported small cluster encompassing phages targeting Roseobacter and Achromobacter hosts. This small phage clade is loosely enclosed in a larger cluster defined primarily by Pseudomonas YuA-like phages (Amgarten et al., 2017), and far removed from the other two main Alphaproteobacteria phage clusters identifiable in the tree: the HTVC019P-like Pelagibacter phages (Zhao et al., 2019) and the ΦCbk-like Caulobacter phages (Gill et al., 2012). A genome-map comparison of vB_BsubS-Delta with members of these three clusters (Figure 4) shows that vB_BsubS-Delta shares only scattered and weak similarity with these disparate groups of phages. Clear hits to Pelagibacter HTVC019P-like and Caulobacter ΦCbk-like phages are restricted to the ribonucleoside-diphosphate reductase subunits alpha and beta (~50 kbp mark in Figure 4), which are known to disseminate laterally among phages (Dwivedi et al., 2013). In contrast, hits to the Pseudomonas YuA-like phages encompass primarily the major capsid and terminase proteins (~10 kbp mark in Figure 4), supporting the notion of an ancient relationship between vB_BsubS-Delta and Pseudomonas YuA-like phages as inferred in Figure 3.

### Distribution of putative CtrA-binding sites in phage genomes

**Figure 5:**
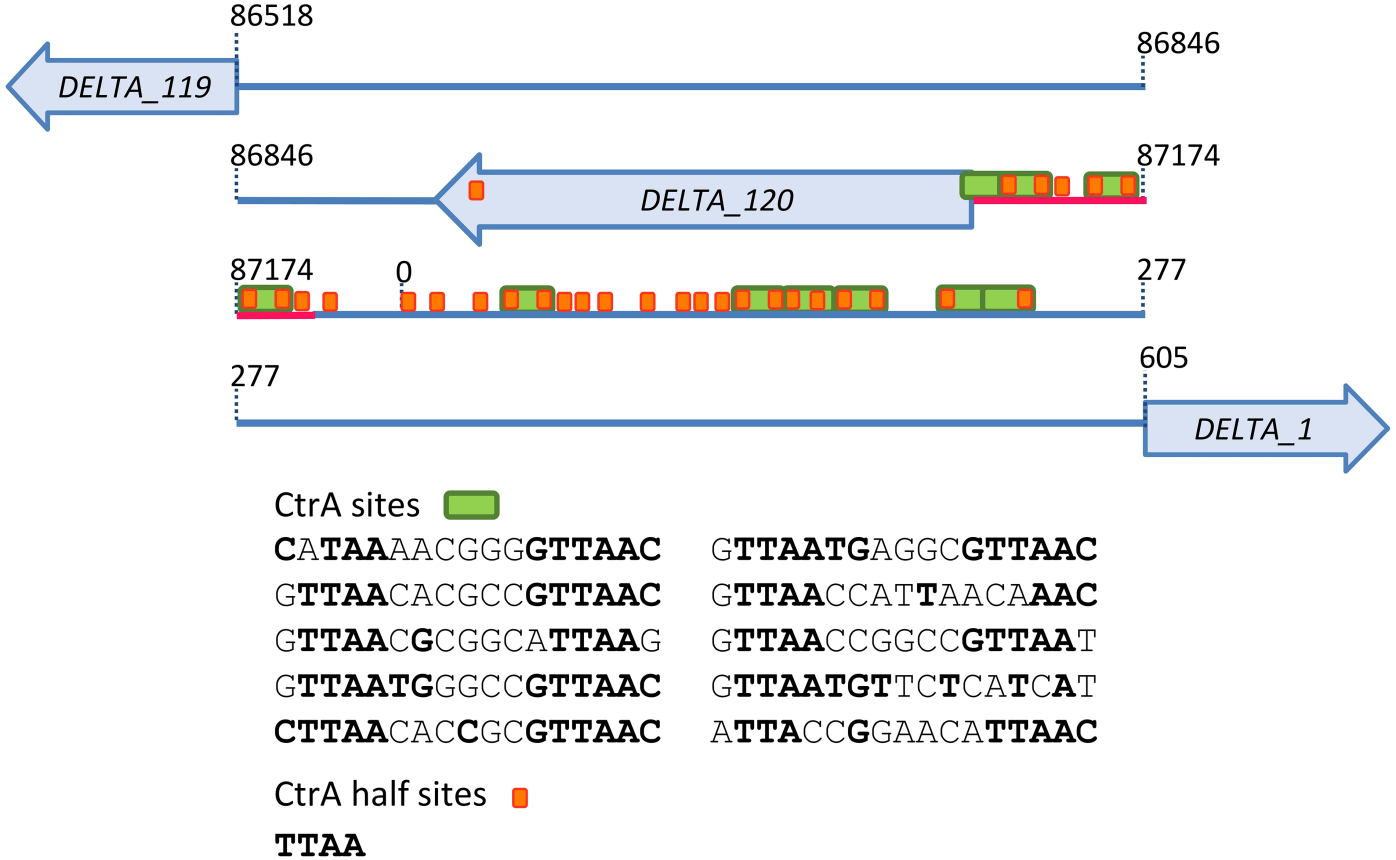
Brevundimonas phage vB_BsubS-Delta gap region diagram. The positions of putative CtrA-binding sites and half-sites in Brevundimonas phage vB_BsubS-Delta gap region are displayed as green and orange boxes, respectively. Their sequences are reported, with consensus-matching bases in bold. The location of protein coding genes, including the predicted DELTA_120 open reading frame, is also shown. The 81 bp probe upstream of DELTA_120 used in electromobility shift assays is underlined in red.

The vB_BsubS-Delta genome contains a relatively large (1,312 bp) gap region separating divergently transcribed genes (Figure 5). Visual inspection of this region revealed the presence of 30 TTAA repeats, 12 of which form canonical TTAA-N7-TTAA CtrA-binding sites following the dyad-spacer CtrA-binding motif consensus (Quon et al., 1996). The TTAA-N7-TTAA CtrA-binding motif has been shown to be conserved and functional across the Alphaproteobacteria, with the dyad elements mediating the specific interaction with CtrA (Barnett et al., 2001; Brilli et al., 2010; Mercer et al., 2010; Greene et al., 2012; Poncin et al., 2018). Further analysis of this region identified a previously undetected open reading frame (hereafter referred to as DELTA_120) predicted to encode a 68 aa protein and located immediately downstream of a cluster of CtrA-binding sites. Homology search with HHpred revealed weak (probability 72–80%) structural similarity of DELTA_120 to several thioltransferases, but no known function could be conclusively assigned to the DELTA_120 products. CtrA regulates cell-cycle control in the Caulobacterales and, more broadly, motility and chemotaxis in the Alphaproteobacteria. The presence of a genomic region with high concentration of CtrA-binding sites in a pilitropic phage therefore suggested that these sites might play a functional role in the vB_BsubS-Delta genome.

To investigate whether this genomic feature was exclusive to vB_BsubS-Delta, we performed a comparative analysis of CtrA-binding site distribution on phage genome sequences. This comparative analysis included 301 complete genome sequences of phages partially related to vB_BsubS-Delta (Supplementary Table S2), 362 complete sequences of Alphaproteobacteria-infecting phages (Supplementary Table S3), and complete sequences from 93 known pilitropic and flagellotropic phages (Supplementary Table S5).

**Figure 6:**
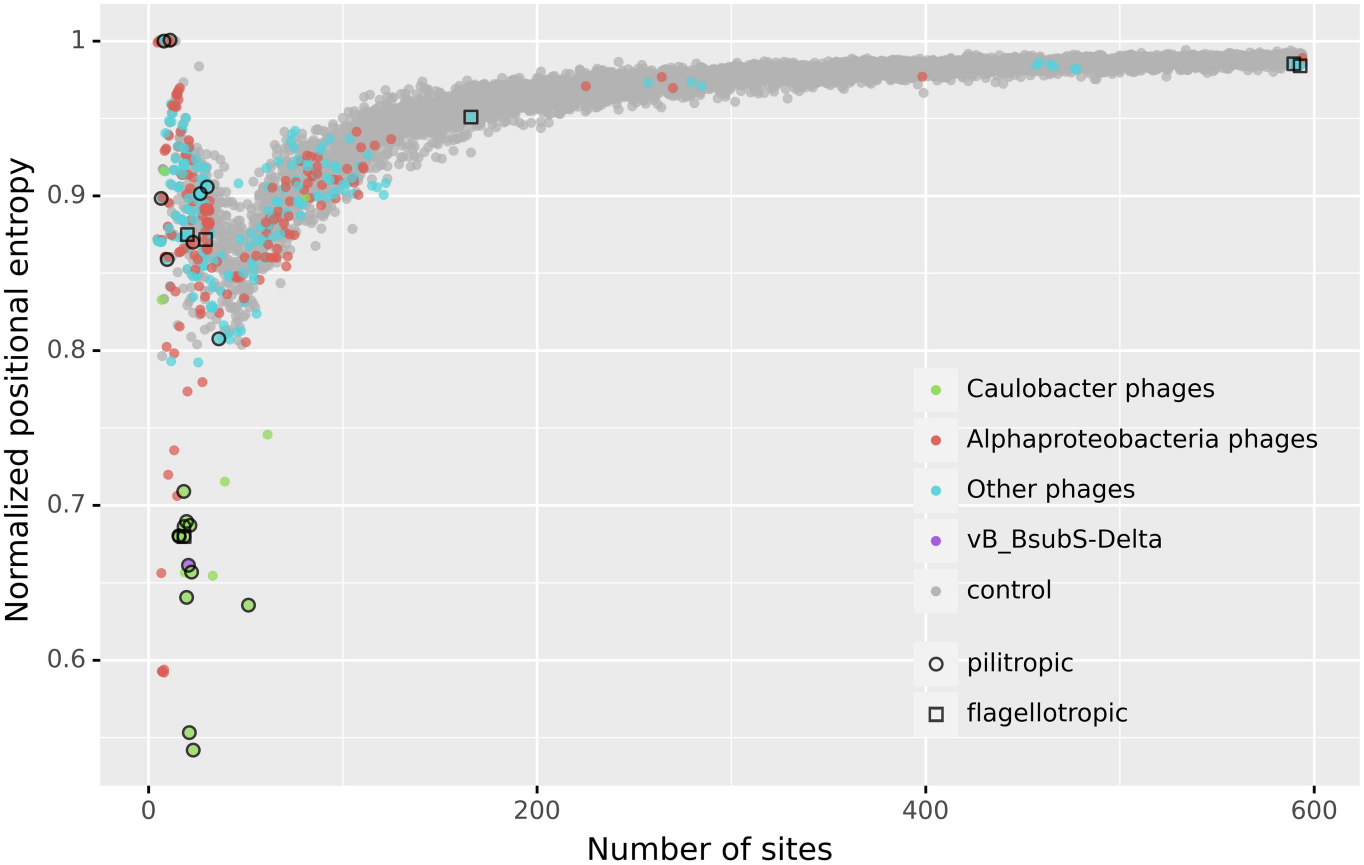
Normalized Positional Entropy vs. number of identified CtrA-binding sites. Each filled circle indicates the normalized positional entropy of the genome as a function of the number of putative binding sites detected in its genome sequence. Uniform jitter was applied for clarity. Color coding in filled circles denotes, respectively, *Caulobacter* phages (green), other Alphaproteobacteria phages (red), vB_BsubS-Delta (purple), all the other analyzed phages (cyan) and the negative control (gray). The negative control was obtained by generating 10 random positional distributions for each possible number of sites and computing their normalized positional entropy (for a total of 5,890 data points). Open circles designate known pilitropic phages, and open squares denote known flagellotropic phages (Supplementary Table S4).

The results shown in Figure 6 reveal that the genomes of a small subset of phages display marked asymmetry in the positional distribution of putative CtrA-binding sites, as measured by positional entropy (Supplementary Tables S6, S7). The same results are obtained when the Gini coefficient, a different measure of distribution inequality, is applied to the positional distribution of putative CtrA-binding sites (Supplementary Figure S2). The plot clearly delineates a set of low positional entropy genome (LPEG) phages with remarkably low normalized positional entropy (Ĥ < 0.75) with regard to both the expected value given the identified number of sites and the rest of phage genomes analyzed. Identical analyses using the known TF-binding motifs of other transcription factors involved in Alphaproteobacteria cell-cycle regulation (GcrA, SciP and DnaA) yielded no evidence of asymmetry in the distribution of putative TF-binding sites for any subset of phage genomes (Supplementary Figure S3).

The LPEG group of phages is dominated by pilitropic *C. crescentus* ΦCbk-like phages, but encompasses also vB_BsubS-Delta and some other Alphaproteobacteria-infecting phages. Several of the LPEG phages branch together with ΦCbk-like phages in Figure 3 and are known to share substantial genomic and morphological similarities with them. In spite of their larger size, Caulobacter phages CcrBL9, CcrBL10, CcrSC and CcrPW share a core proteome of 69 proteins with ΦCbk-like phages (Wilson and Ely, 2019). The remaining LPEG phages present little sequence similarity with previously sequenced phages (Figure 3). Like vB_BsubS-Delta, Sphingomonas phage vB_StuS_MMDA13 is a siphophage that has been reported to contain a large intergenic gap separating divergently transcribed blocks of genes (Marmo et al., 2020, 13). In contrast, Rhizobium phage Pasto, presumed to be temperate, is a podophage with short DTRs (Manuel et al., 2021). Rhizobium phage Palo is an unrelated podophage infecting *Rhizobium phaseoli* with short DTRs (Nabhani et al., 2021). Ruegeria phage DSS3Φ1 and closely related phages (vB_RpoS-V7, vB_RpoS-V11, vB_RpoS-V18, vB_RpoS-V16) have no large intergenic gaps or reported DTRs and are closely related to flagellotropic phages of the Chivirus genus. These phages encode a repressor/integrase tandem and are presumed to be temperate phages (Zhan et al., 2018).

### Properties of CtrA-binding sites in LPEG phages

**Figure 7:**
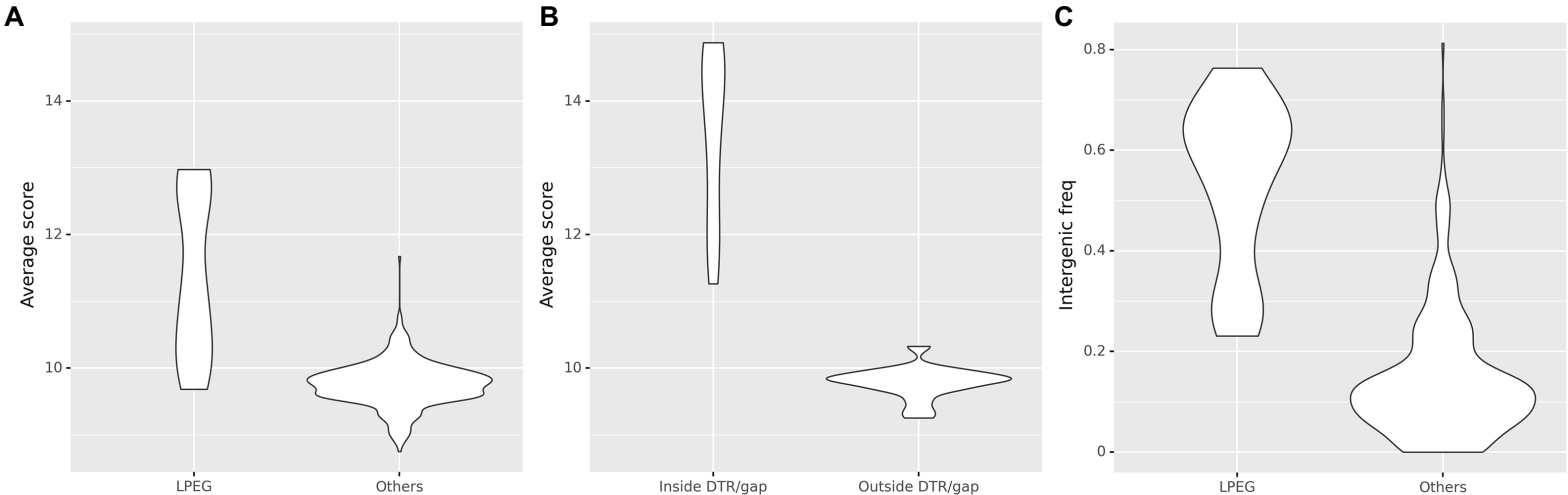
Comparison of predicted CtrA-binding sites between LPEG and control phages. **(A)** Comparison of the distribution of the average PSSM score for predicted CtrA-binding sites over the entire genome, in LPEG phages and all the other analyzed phage genomes. **(B)** Comparison of the distribution of the average PSSM score for predicted CtrA-binding sites identified within and outside DTR/gap regions in LPEG phage genomes. **(C)** Comparison of the distribution of intergenic frequency for predicted CtrA-binding sites (defined as the proportion of predicted CtrA-binding sites identified in non-coding regions) in LPEG and all the other analyzed phage genomes.

To elucidate the possible role of the CtrA-binding sites in LPEG phages, we analyzed other properties of identified CtrA-binding sites both within high-density regions and overall in the genome. The PSSM score is a well-established indicator of binding specificity for most transcription factors (Zhao and Stormo, 2011). The average score for CtrA-binding sites in these phages is substantially larger than in control phages (Mann–Whitney U test *p* < 10^−5^; Figure 7A). Furthermore, the majority of putative CtrA-binding sites in the genome sequences of LPEG phages colocalizes to the distal ends of these phages, where DTRs or gap regions have been primarily reported (Supplementary Figure S4), and the average score of sites in DTRs and gap regions is larger than in the rest of the genome (Mann–Whitney U test p < 10^−5^; Figure 7B). These differences are also observed when comparing LPEG phages to Alphaproteobacteria-infecting phages (Supplementary Figure S5), and indicate that the CtrA-binding sites in LPEG phage genomes, and particularly in their DTR and gap genomic regions, may be under positive selection for CtrA binding.

**Figure 8:**
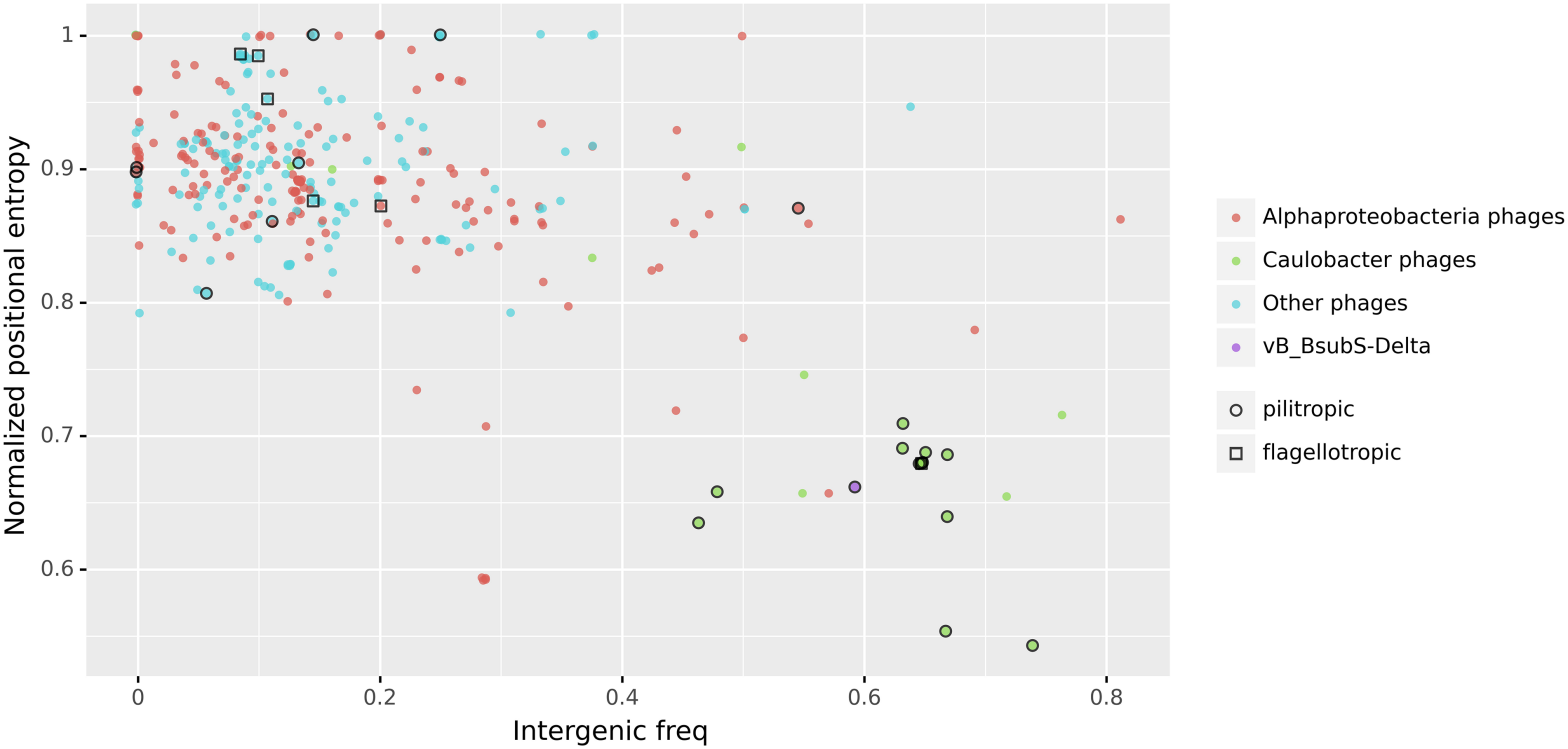
Normalized Positional Entropy vs. intergenic frequency of identified CtrA-binding sites. Each filled circle indicates the normalized positional entropy of the genome as a function of the intergenic frequency of putative CtrA-binding sites detected in its genome sequence. Uniform jitter was applied for clarity. Color coding in filled circles denotes, respectively, *Caulobacter* phages (green), phages infecting other Alphaproteobacteria (red), vB_BsubS-Delta phage (purple) and all the other analyzed phages (cyan). Open circles designate known pilitropic phages, and open squares denote known flagellotropic phages (Supplementary Table S4).

Putative CtrA-binding sites in LPEG phage genome sequences also localize primarily to intergenic regions (Mann–Whitney U test p < 10^−5^; Figure 7C), and this effect is mostly driven by the high-scoring sites in DTRs and gap regions (Supplementary Figure S6). This suggests that, if bound by CtrA, the identified CtrA-binding sites may have regulatory effects on nearby genes. To further characterize the prevalence of intergenic localization for CtrA-binding sites among LPEG phages, we analyzed the distribution of positional asymmetry of predicted CtrA-binding sites as a function of their intergenic localization (Figure 8).

The results show that, when considered together, the genomic concentration and intergenic localization of predicted CtrA-binding sites define a clear separation between LPEG phages and the rest of phages in the dataset. The new analysis indicates that two additional Alphaproteobacteria-infecting phages also present an unusual distribution of predicted CtrA-binding sites (Supplementary Table S7). Sphingobium phage Lacusarx (Ĥ = 0.78) and Mesorhizobium phage Cp1R7A-A1 (Ĥ = 0.77) are both siphophages with previously reported morphological and genomic similarities with ΦCbk-like phages. These similarities encompass similar tail decorations, orthologous replication module and tRNA clusters, the presence of large DTRs and a postulated virulent lifestyle (Nielsen et al., 2017; Gunathilake et al., 2021). The presence of a distinct genomic signature in the predicted CtrA-binding site distribution of these phages, with weaker colocalization than observed in LPEG phages but with strong preference for intergenic regions, suggests that the hypothetical transcriptional role of identified CtrA-binding sites in Alphaproteobacteria-infecting phages may extend to non-LPEG phages.

### Properties of genomic regions with high density of putative CtrA-binding sites

**Figure 9:**
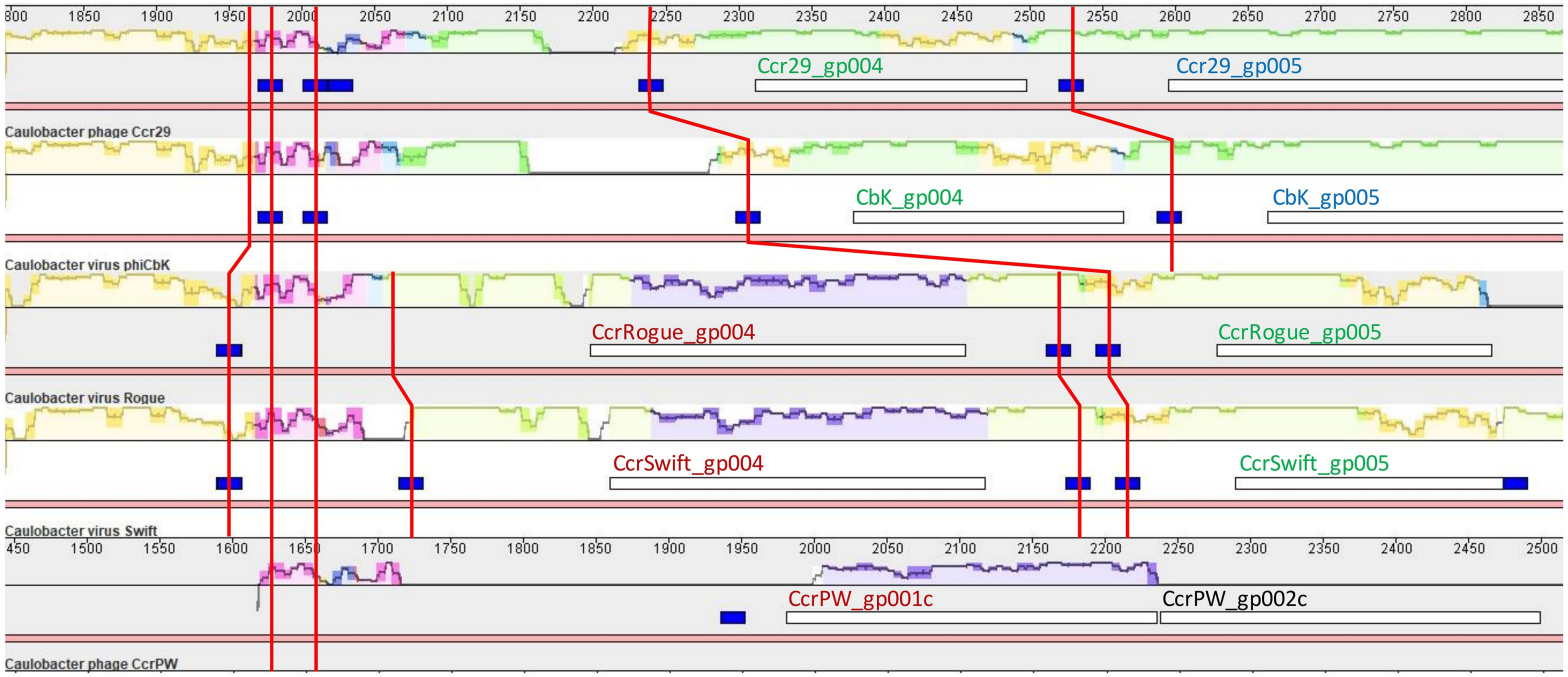
Partial view of Mauve alignment of DTRs from Caulobacter ΦCbk-like phages with significant sequence identity. Sequence conservation is shown using the backbone color scheme, with mauve regions representing areas conserved among all displayed genomes. Open reading frames are shown as white boxes and predicted CtrA-binding sites as blue boxes. Superimposed red lines trace the conserved regions harboring predicted CtrA-binding sites. Colors in the locus tags of open reading frames designate orthologous genes.

Sphingobium phage Lacusarx and Mesorhizobium phage Cp1R7A-A1 define a well-supported phylogenetic cluster with ΦCbk-like phages (Figure 3). These phages all present large DTRs with a high concentration of predicted CtrA-binding sites and multiple protein coding genes with no known function. To investigate the evolutionary origin of the high-prevalence of predicted CtrA-binding sites in DTR regions, we performed multiple sequence alignment of DTRs for representative members of the different ΦCbk-like subclusters using Mauve and computed pairwise sequence identities with ViPhy. The resulting alignment (Supplementary Figure S7) revealed that the DTRs of Caulobacter phages Ccr29, ΦCbk, Rogue and Swift present significant structural similarities and substantial pairwise DNA sequence identity [ranging from 90 to 47% (Supplementary Table S8)], as expected from previous genomic analyses (Gill et al., 2012). The DTRs of these phages present only low sequence identity to the Caulobacter phage CcrPW (12%) and Caulobacter phage CcrSC (8%) DTRs. The DTRs of Sphingobium phage Lacusarx and Mesorhizobium phage Cp1R7A-A1 present marginal pairwise sequence identity (1%), and similar residual similarity to Caulobacter phage CcrSC. Even among the closely related Caulobacter ΦCbk-like phages (Ccr29, ΦCbk, Rogue and Swift), predicted CtrA-binding sites are not fully conserved, and there is evidence of gain/loss of CtrA-binding sites upstream of orthologous genes following genomic rearrangements in DTRs (Figure 9). No known function could be conclusively assigned, based on homology search, to the product of any of the genes in these DTRs presenting predicted CtrA-binding sites in their upstream region.

The remaining phages harboring regions with a high concentration of CtrA-binding sites reveal unique configurations. Predicted CtrA-binding sites in Rhizobium phage Pasto are primarily located toward the end of its DTR, which shows no significant similarity to any other sequenced genomes (Manuel et al., 2021), and extend to the upstream region of the first Pasto gene (CPT_Pasto_001), which encodes a Bacillus phage Φ29 single-stranded DNA-binding protein homolog (Supplementary Figure S8). In Rhizobium phage Palo, intergenic CtrA-binding sites are found following its DTR, in the promoter region of genes CPT_Palo_014 and CPT_Palo_015, which have no known function (Supplementary Figure S8). Like vB_BsubS-Delta, Sphingomonas phage vB_StuS_MMDA13 contains a ~ 1 Kb gap region separating divergently transcribed blocks of genes (Marmo et al., 2020). This region was shown to contain four instances of an imperfect 12 bp direct repeat motif (5′-T_3_G_2_C_4_G_4_T_4_T_4_A_4_A_4_C_4_C_4_A_3_T_4_–3′) upstream of gene MMDA13_gp54, which has no known function. These direct repeat motif instances overlap with the three putative CtrA-binding sites identified here (Supplementary Figure S9). In contrast to other LPEG phages, Ruegeria phage DSS3Φ1 and closely related phages lack DTRs or gap regions, and present weak localization of CtrA-binding sites to intergenic regions (Supplementary Figure S10). Predicted CtrA-binding sites also have consistently lower scores (10.07 ± _SD_ 0.10) than those of other LPEG phages, and their concentration in the genome is largely driven by three intragenic sites in gene DSS3P1_09 and its orthologs in the other Ruegeria phages.

The analysis of genomic regions with high density of predicted CtrA-binding sites hence indicates that such regions have independently evolved at least five times in unrelated Alphaproteobacteria-infecting phages (the ΦCbk-like cluster, vB_BsubS-Delta, Sphingomonas phage vB_StuS_MMDA13, Rhizobium phage Palo and Rhizobium phage Pasto). Moreover, the high concentration of CtrA-binding sites in these regions appears to have been maintained in the face of substantial genomic divergence and reorganization within the diverse ΦCbk-cluster, which encompasses phages infecting three different bacterial genera (Caulobacter, Sphingobium and Mesorhizobium). Together, the consistent concentration of putative CtrA-binding sites in the specific genomic regions, their localization in intergenic space and the fact that this pattern is only observed in Alphaproteobacteria-infecting hosts that encode a functional CtrA protein strongly suggests that the colocalization of CtrA-binding sites in these phage genomes has a functional role involving CtrA binding.

### CtrA binds specifically to predicted CtrA-binding sites in LPEG phages

We performed Electrophoretic Mobility Shift Assays (EMSA) to experimentally determine whether CtrA binds to predicted CtrA-binding sites or not. After unsuccessful attempts at purifying *B. subvibrioides* CtrA, *C. crescentus* CtrA was purified from *E. coli* using an expression construct. *B. subvibrioides* and *C. crescentus* are closely related, both belonging to the Caulobacterales order (Curtis and Brun, 2014; Adhikari et al., 2021), and BLASTP showed that these proteins are 87% identical. A multiple sequence alignment revealed that representative Alphaproteobacteria CtrA homologs present an average amino acid identity of 74.44% ± _SD_ 11.13, and confirmed that the *B. subvibrioides* CtrA contains all the domains and amino acid residues critical to its response regulator activity, including the well-documented *C. crescentus* 51D phosphorylation site, the acidic pocket active site and the OmpR-like DNA-binding domain (Quon et al., 1996; Barnett et al., 2001; Supplementary Figure S11). Heterologous expression of CtrA orthologs from Alphaproteobacteria distantly related to *C. crescentus* [e.g., *Sinorhizobium meliloti* (82.25% identity), *Rickettsia prowazekii* (58.01% identity)] have been shown to fully complement a *C. crescentus* CtrA mutant (Barnett et al., 2001; Brassinga et al., 2002), confirming that the CtrA phosphorelay machinery and the CtrA-binding motif are conserved and functional across the Alphaproteobacteria (Brilli et al., 2010). CtrA is a response regulator that preferentially binds to DNA in its phosphorylated form. To overcome this issue, we phosphorylated CtrA *in vitro* prior to the EMSA using a modified form of a previously described methodology (Reisenauer et al., 1999).

Binding of CtrA was assessed using DNA from three different LPEG phages. For Brevundimonas phage vB_BsubS-Delta, a region of dsDNA (81 bp) upstream from the start codon of DELTA_120 was used as a probe, as this was the open reading frame with its 5′ end closest to the high-density region of predicted CtrA-binding sites. This DNA probe included three CtrA-binding sites and two half-sites (Figure 5). For Caulobacter phage ΦCbk, 99 and 114 bp regions upstream of genes CbK_gp005 and CbK_gp018, both containing one predicted CtrA-binding site, were used as probes. For Mesorhizobium phage Cp1R7A-A1, a 123 bp region upstream of gene Cp1R7AA1_019, containing a single putative CtrA-binding site was used as a probe. No known function could be assigned to any of these genes *via* homology search.

**Figure 10:**
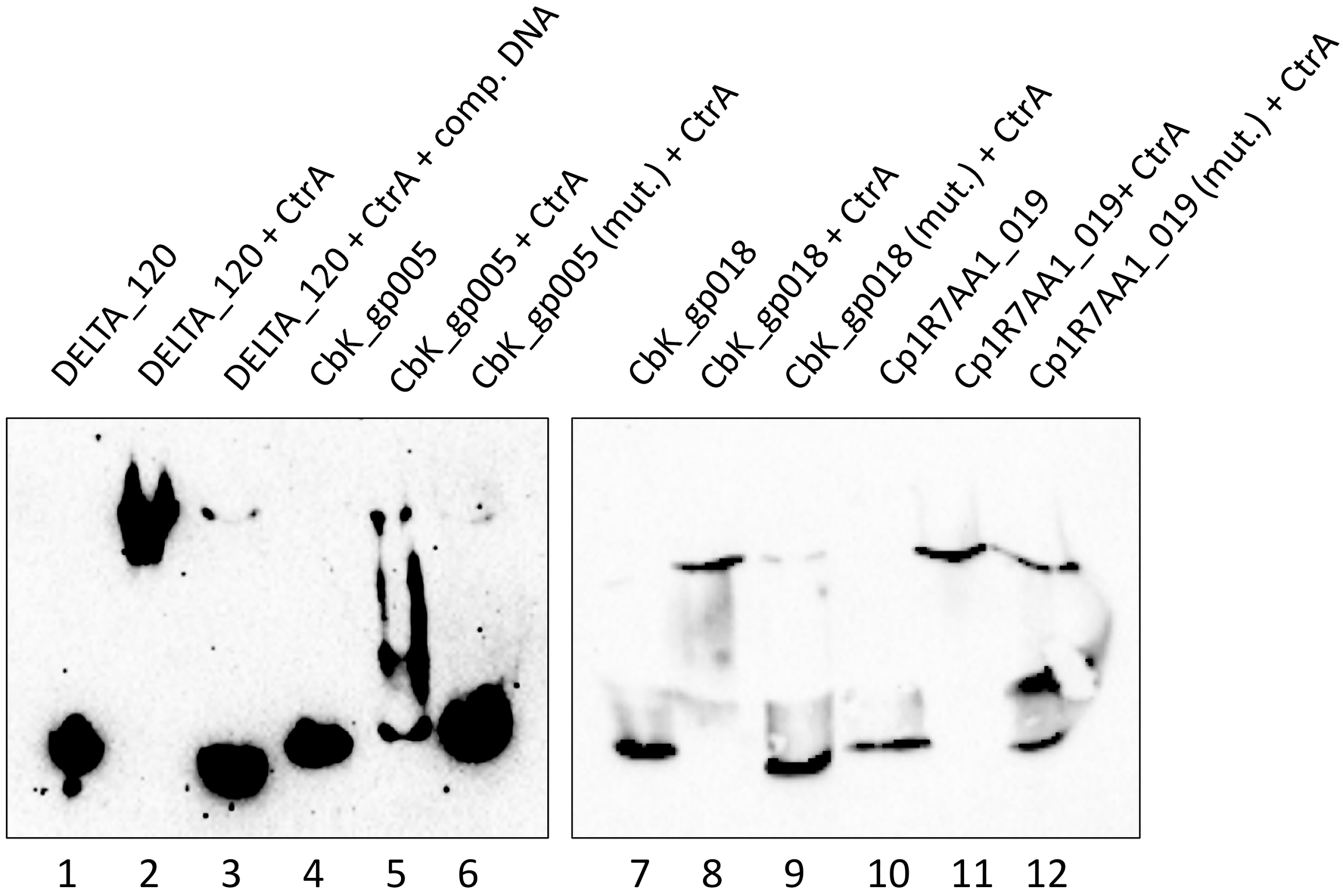
CtrA binds to LPEG phage DNA containing predicted CtrA-binding sites. EMSA assays using purified *C. crescentus* CtrA protein and DNA probes from different LPEG phages. Lane 1: Brevundimonas phage vB_BsubS-Delta DELTA_120 DNA. Lane 2: DELTA_120 DNA with phosphorylated CtrA. Lane 3: DELTA_120 DNA with phosphorylated CtrA and unlabeled DELTA_120 competitor DNA at 100-fold molar excess. Lane 4: Caulobacter phage ΦCbk CbK_gp005 DNA. Lane 5: CbK_gp005 DNA with phosphorylated CtrA. Lane 6: CbK_gp005 DNA with mutated CtrA binding site, with phosphorylated CtrA. Lane 7: Caulobacter phage ΦCbk CbK_gp018 DNA. Lane 8: CbK_gp018 DNA with phosphorylated CtrA. Lane 9: CbK_gp018 with mutated CtrA binding site, with phosphorylated CtrA. Lane 10: Mesorhizobium phage Cp1R7A-A1 Cp1R7AA1_019 DNA. Lane 11: Cp1R7AA1_019 DNA with phosphorylated CtrA. Lane 12: Cp1R7AA1_019 DNA with mutated primary CtrA binding site, with phosphorylated CtrA.

As shown in Figure 10, the vB_BsubS-Delta DELTA_120 DNA probe by itself migrates near the bottom of the gel (lane 1), but when the phosphorylated CtrA protein is added a significant mobility shift is observed (lane 2). In addition, when CtrA was incubated with unlabeled probe at 100-fold excess before incubating with labeled probe, most of the labeled probe was not shifted (lane 3). Similarly, the ΦCbk CbK_gp005 and CbK_gp018 probes shift only in the presence of CtrA (lanes 5 and 8), but not in its absence (lanes 4 and 7). In both cases, the CtrA-induced retardation shift is abolished when a probe containing mutations in the predicted CtrA-binding site is used. Identical results are obtained for the Cp1R7A-A1 Cp1R7AA1_019 probe (lanes 10, 11 and 12). The lower molecular weight shift observed in lane 12 is likely the result of CtrA binding to two orphan TTAA half-sites that were not mutated in that probe. Overall, these results show that CtrA binds specifically to predicted CtrA-binding sites in non-orthologous regions from three different phages infecting distinct Alphaproteobacteria hosts.

### CtrA regulates gene expression in LPEG phages

**Figure 11:**
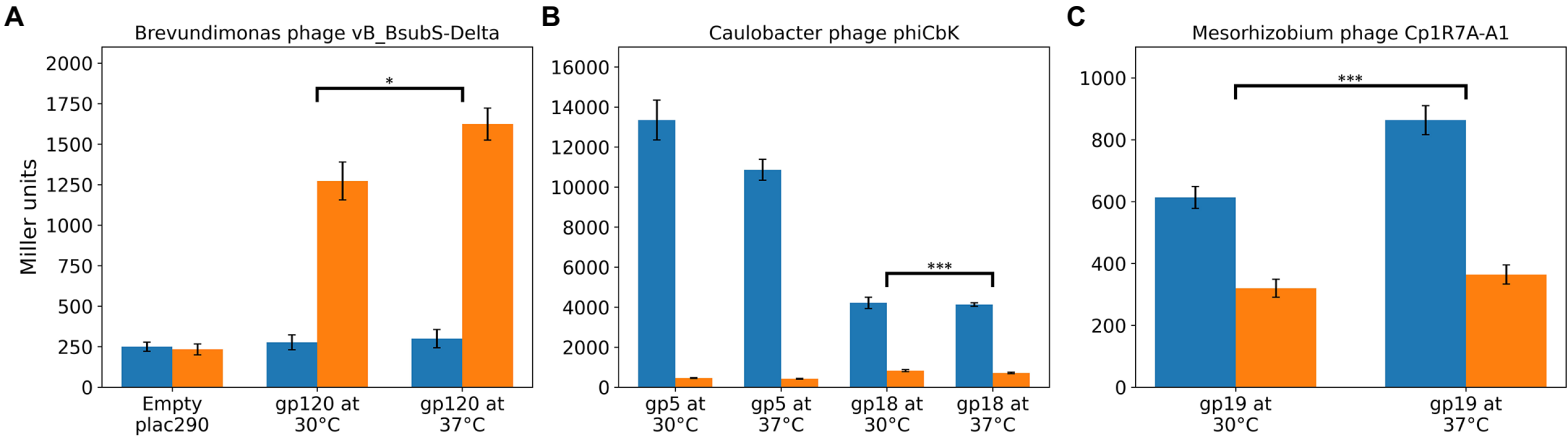
CtrA regulates gene expression in multiple LPEG phages. Standardized amount of β-Gal activity for *C. crescentus* cells harboring a plac290 vector containing the promoter region of target genes fused with a promoter-less beta-galactosidase gene. Blue and orange denote, respectively, results for the WT and temperature-sensitive CtrA LS2195 *C. crescentus* strains. **(A)** Expression levels for Brevundimonas phage vB_BsubS-Delta DELTA_120 genes. **(B)** Expression levels for Caulobacter phage ΦCbk constructs for genes CbK_gp005 (gp5) and CbK_gp018 (gp18). **(C)** Expression levels for Mesorhizobium phage Cp1R7A-A1 Cp1R7AA1_019 (gp19) gene. Stars designate statistical significance (one-sided Mann–Whitney U test) in the difference between log-fold change (WT vs. LS2195) values at 30 and 37°C.

To determine if CtrA binding affected expression of genes with upstream regions bound by CtrA, we created reporter constructs in which the promoter regions of DELTA_120 (Supplementary Figure S12), CbK_gp005 and CbK_gp018, and Cp1R7AA1_019 (Supplementary Figure S13) were fused with a promoter-less beta-galactosidase gene in the plac290 plasmid (Gober and Shapiro, 1992). Given that *ctrA* is essential in many Alphaproteobacteria, including *B. subvibrioides* (Curtis and Brun, 2014), a *ctrA* null strain cannot be constructed. However, LS2195 is a *C. crescentus* strain with a temperature-sensitive allele of CtrA; the T401I mutation confers viability at 30°C but is lethal at 37°C due to CtrA inactivation (Quon et al., 1996). Given that *C. crescentus* CtrA had been shown to bind to the sequences of the expression constructs (Figure 10), we examined the expression of the expression constructs in *C. crescentus* NA1000 (WT) and LS2195 (temperature sensitive) strains (Figure 11).

In the WT *C. crescentus* strain, vB_BsubS-Delta DELTA_120 construct expression was similar to the empty vector at both temperatures. However, when the construct was examined in the temperature-sensitive strain at the permissive temperature, expression was >4.5-fold higher (Figure 11A). Under the assumption of CtrA regulation, a shift is expected, since it is known that the temperature-sensitive CtrA strain has compromised CtrA function even at the permissive temperature, which then becomes exacerbated to non-viability at the restrictive temperature (Quon et al., 1996; Domian et al., 1999). This suggests that compromised CtrA function leads to an increase in DELTA_120 gene expression. When the strain was shifted to the restrictive temperature for 1 h, expression further increased to almost 6-fold higher than WT (Figure 11A), a shift that is larger than that observed for other CtrA-regulated genes (Domian et al., 1999). This data, combined with the EMSA results (Figure 10), suggests that CtrA binds to the vB_BsubS-Delta phage genome and represses DELTA_120 expression.

The expression results for Caulobacter phage ΦCbk (CbK_gp005 and CbK_gp018) and Mesorhizobium phage Cp1R7A-A1 (Cp1R7AA1_019) stand in stark contrast to those observed in vB_BsubS-Delta. The two Caulobacter phage ΦCbk constructs showed robust expression in WT *C. crescentus* (Figure 11B), with the CbK_gp005 construct showing the highest expression. Conversely, both constructs showed virtually no expression in the LS2195 strain at both the permissive and restrictive temperatures. Expression levels for the CbK_gp005 and CbK_gp018 constructs were, respectively, >29-fold and 5-fold lower in the LS2195 strain compared to WT at the permissive temperature. Similar results were observed for the Cp1R7AA1_019 promoters from Mesorhizobium phage Cp1R7A-A1. Expression from this construct was almost 2-fold lower in the LS2195 strain at the permissive temperature compared to WT strain (Figure 11C). As with the previous constructs, the expression levels were similar when the LS2195 strain was shifted to 37°C for 1 h. The fact that CtrA binds to predicted CtrA-binding sites in these promoters (Figure 10) and these results showing CtrA-dependent transcription suggest that CtrA acts as an activator for Caulobacter phage ΦCbk and Mesorhizobium phage Cp1R7A-A1 genes.

## Discussion

### Independent evolution of gene regulation by host CtrA in multiple phage genomes

A systematic analysis of the distribution of predicted CtrA-binding sites in 499 phage genome sequences uncovered an unequivocal genomic signature in several Alphaproteobacteria-infecting phages, here termed LPEG phages (Figure 6). These phages present marked positional asymmetry in the distribution of predicted CtrA-binding sites, which localize primarily to either intergenic gap regions or direct terminal repeats in these phage genomes. Furthermore, putative CtrA-binding sites in LPEG phages present both higher scores and preferential localization to intergenic regions, with both traits being driven largely by CtrA-binding sites in their DTRs or gap regions (Figure 7). The correlation between genomic positional asymmetry and intergenic localization (Figure 8) led to the identification of additional phages with unusual CtrA-binding site distribution. CtrA-binding sites often colocalize with DnaA- and SciP-binding sites in *C. crescentus* (Tan et al., 2010; Taylor et al., 2011), but no evidence of positional asymmetry was found in the distribution of predicted binding sites for DnaA, SciP or the functionally related GcrA transcription factor, irrespective of the threshold used to identify putative binding sites (Supplementary Figure S3). The functional role of predicted CtrA-binding sites was experimentally determined by EMSA and β-Gal assays in Brevundimonas phage vB_BsubS-Delta, Caulobacter phage ΦCbk and Mesorhizobium phage Cp1R7A-A1 (Figures 10, 11). Phosphorylated CtrA was found to bind promoter regions containing putative CtrA-binding sites and to repress (vB_BsubS-Delta) or activate (ΦCbk & Cp1R7A-A1) transcription of the associated genes.

Phylogenetic analysis of Alphaproteobacteria-infecting phages and phages potentially related to Brevundimonas phage vB_BsubS-Delta revealed that LPEG phages map to ostensibly unrelated phage groups, with vB_BsubS-Delta branching deep within a cluster encompassing Pseudomonas YuA-like phages, Ruegeria phages defining a deep independent branch, Sphingomonas phage vB_StuS_MMDA13 in an heterogeneous cluster encompassing phages infecting different Proteobacteria classes, and the rest of LPEG phages mapping to a large cluster of ΦCbk-like phages (Figure 3). The vast phylogenetic distance between these disparate groups of phages, with no detectable nucleotide sequence identity (Supplementary Table S9), indicates that the presence of colocalizing, predominantly intergenic CtrA-binding sites has evolved independently in all these phage lineages. Even among the Caulobacter ΦCbk-like phages, the DTRs harboring predicted CtrA-binding sites often share scant nucleotide sequence identity, and a multiple sequence alignment of DTR regions reveals substantial turnover in putative CtrA-binding sites among closely related phages (Figure 9; Supplementary Figure S7). This, together with the experimental observation of distinct modes of CtrA activity in promoters from different phages (Figure 11), indicates that the prevalence of CtrA-binding sites in these regions has been actively maintained in the face of substantial variation and rearrangement in these regions.

A number of LPEG phages are known, or suspected based on substantial genomic similarity, to be pili/flagellotropic. The best known example, Caulobacter phage ΦCbk, has been shown to target flagella for initial attachment, followed by subsequent adsorption at pilus portals (Guerrero-Ferreira et al., 2011). CtrA is best known for its role in cell-cycle progression in the Rhizobiales, but experimental analyses indicate that the ancestral role of CtrA in the Alphaproteobacteria is the regulation of motility, chemotaxis and some quorum sensing components (Bird and MacKrell, 2011; Greene et al., 2012; Zan et al., 2013, 1; Wang et al., 2014; Francez-Charlot et al., 2015; Hernández-Valle et al., 2020), and a comparative genomics analysis of representatives from all Alphaproteobacteria orders supports this observation (Supplementary Figure S14). The finding that CtrA regulates gene expression in several pili/flagellotropic phages raises the tantalizing possibility that different groups of phages may have independently evolved gene regulation mechanisms dependent upon the same transcriptional regulator that governs the generation of the cellular appendages required for efficient phage infection.

### CtrA-mediated lytic deferment in Alphaproteobacteria-infecting phages

Decision-making in bacteriophages revolves predominantly around the control of the phage lytic cycle in response to external stimuli. Enterobacteria phage Lambda and Salmonella phage P22 are canonical temperate phages encoding transcriptional repressors that activate the lytic cycle in response to DNA damage (Phizicky and Roberts, 1980, 22), and several unrelated groups of phages have independently evolved the ability to activate their lytic cycle leveraging the host transcriptional repressor LexA, which responds to DNA damage using the same molecular mechanism as phage repressors (Sassanfar and Roberts, 1990; Shearwin et al., 1998; Kimsey and Waldor, 2009; Kim and Ryu, 2013; Fornelos et al., 2015). Conversely, phages of the Φ29 family exploit the Spo0A sporulation master regulator to halt the lytic cycle if the host transitions into the sporulation pathway (Castilla-Llorente et al., 2009). This allows spore entrapment of the phage chromosome, enabling the phage to attain a dormant state in the endospore (Meijer et al., 2005). These examples illustrate that the availability of a reliable signal transduced by a cellular transcription factor can be readily exploited by phages to control their lytic cycle.

**Figure 12:**
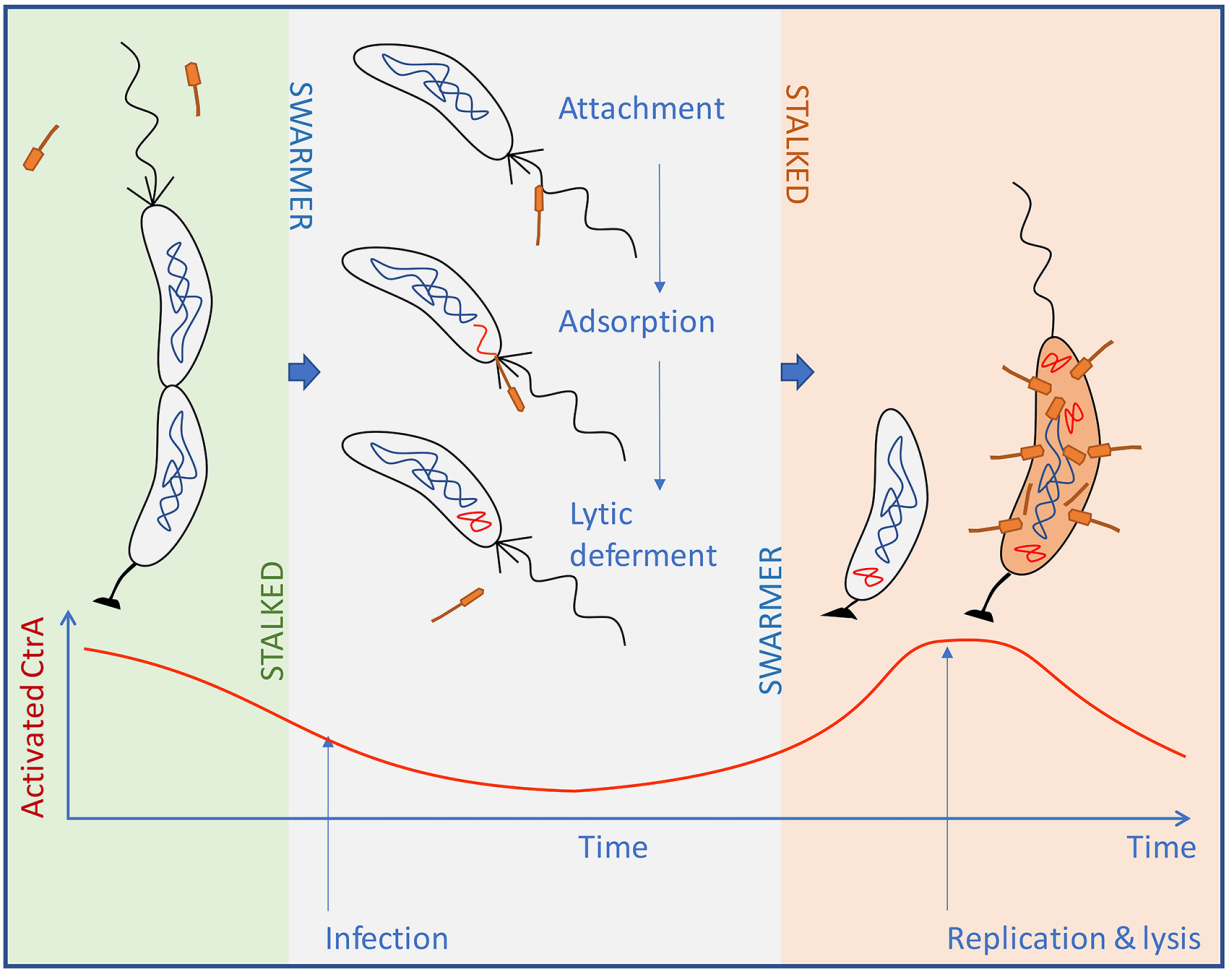
Schematic representation of the proposed life cycle for ΦCbk-like phages. Phages infecting *Caulobacter*-like species require the pili/flagella present in swarmer cells for infection. Upon infection, cells encounter non-peak levels of activated CtrA and defer the lytic cycle. After the swarmer cell transitions to a stalked cell, levels of activated CtrA start to rise, eventually triggering the expression of phage genes and the development of the lytic cycle in an environment where swarmer cells are being produced.

Our results indicate that several groups of pili/flagellotropic Alphaproteobacteria-infecting phages have convergently evolved the ability to use the cellular CtrA transcription factor to regulate the expression of a small set of uncharacterized genes. This suggests that there is a clear adaptive advantage to utilizing CtrA in phage decision-making. We hypothesize that pili/flagellotropic Alphaproteobacteria-infecting phages leverage the host CtrA to implement a form of lytic deferment, conceptually similar to that observed in Bacillus Φ29-like phages. We illustrate this hypothesis on the well-studied Caulobacter-ΦCbk system (Figure 12), as a representative of the Caulobacterales and Hyphomicrobiales orders. In these orders, CtrA acquired a well-defined role as global regulator and key component of cell cycle progression, which subsequently tied flagellum and pilus synthesis to specific stages of the cell cycle. In Caulobacter-like bacteria, flagella are synthesized in the predivisional cell stage but rotation is not activated until cell division; pilus filaments are also not synthesized until cell division. Both processes are dependent on CtrA activity. Nearly identical mechanisms are found in members of the Hyphomicrobiales, with cell cycle-driven CtrA-dependent flagellum and/or pilus production seen in *Agrobacterium tumefaciens* (Kahng and Shapiro, 2001) and *S. meliloti* (Xue and Biondi, 2019). This means that for most organisms in the Caulobacterales and Hyphomicrobiales, a flagellotropic/pilitropic phage will only infect swarmer cells.

Moreover, in the infected swarmer cell, the CtrA-regulated phage genes would presumably be repressed, mirroring the silencing of host genes by CtrA in this phase. Thus, CtrA-regulated genes would need the cell cycle of the host to progress before expression could occur. For CtrA-repressed genes (as in Brevundimonas phage vB_BsubS-Delta) this would occur during the swarmer to stalked cell transition when CtrA is cleared from the cell. This event is coincident with host chromosome replication initiation (Quon et al., 1998). For CtrA-induced genes (such as those found in ΦCbk-like phages), the host cell cycle would need to progress through swarmer cell differentiation into the mid-to-late predivisional cell stage, where CtrA is resynthesized and activated by phosphorylation; this is coincident with significant progression of chromosome replication (Domian et al., 1997).

The timing of phage gene expression to host cell cycle progression could provide several advantages. First, members of the *Caulobacter* clade are known to live in oligotrophic environments where nutrients are scarce. Timing phage gene expression to the host cell cycle, particularly stages of the cell cycle that have chromosome replication, may ensure that the host has enough nutrients to synthesize new phage components. Secondly, members of the *Caulobacter* clade are known to often live in multicellular arrangements, either attached to surfaces as biofilms or as multi-cell clusters known as rosettes (Poindexter, 1964). Production of the infectious structures of flagella and pili occurs on swarmer cells. Hence, it is in the phage’s best interest to be around cells that are producing new swarmer cells. This would not be when a swarmer cell is swimming in bulk water, but when a swarmer cell attaches to a colony of cells and differentiates.

We suggest that this CtrA-mandated temporary repression of the phage infection cycle be termed lytic deferment (LD). Although gene regulation is involved, LD is completely distinct from the temperate phage pathway to lysogeny. In lysogeny, the phage infection cycle undergoes a binary choice, toward repression and establishment of the prophage state or toward productive lysis. This choice is influenced by physiological conditions and stochastic events (Arkin et al., 1998), and the frequency of lysogeny varies dramatically between different temperate phages under the same conditions. In contrast, we posit that every ΦCbk infection enters the LD state, which lasts until the host transitions into the stalked cell state. It is also possible that some phage genes may accelerate the transition; indeed, ΦCbk encodes a homolog of GcrA (Gill et al., 2012). We also would make the distinction between LD and the “pseudolysogeny” state that has been reported for various types of phage infections (Łoś and Węgrzyn, 2012). As noted by Łoś et al., pseudolysogeny has not been formally defined in terms of genes that are required for it in any system; rather, the claim for a pseudo-lysogenic state is usually based on the persistence of phage virions and/or gDNAs in a culture that does not undergo lysis. This is clearly different from what we propose is LD, where lysis inevitably occurs as part of the lytic deferment pathway.

We note that lytic deferment would provide time for the swarmer cell to attach to a colony of growing cells, effectively turning the infected swarmer cell into a Trojan Horse (Figure 12). Importantly, LD would be difficult to observe in laboratory conditions. In an actively growing culture, cells are constantly progressing through the cell cycle, and hence rapidly terminating the lytic cycle deferment in the infecting phage. While these are only hypotheses, it is becoming increasingly evident that phage infecting members of the Caulobacterales and Hyphomicrobiales may have an interest in the cell cycle of their hosts. ΦCbk-like phages infecting *Caulobacter* contain homologs of the cell cycle regulator GcrA in their genomes (Gill et al., 2012), and some Sinorhizobium phages carry analogs of the DNA methyltransferase CcrM, which also participates in cell cycle-based gene expression (Decewicz et al., 2017).

Outside the Caulobacterales and Hyphomicrobiales, CtrA is not known to be involved in cell-cycle regulation. However, CtrA has an ancestral role as a regulator in motility, chemotaxis and quorum sensing systems across the Alphaproteobacteria (Bird and MacKrell, 2011; Greene et al., 2012; Zan et al., 2013, 1; Wang et al., 2014; Francez-Charlot et al., 2015; Hernández-Valle et al., 2020). CtrA-mediated lytic deferment would hence provide a significant adaptive advantage to pili/flagellotropic phages infecting the Alphaproteobacteria, enabling phages to either infer the presence of infectable cells in their environment or to sense a loss of motility typically associated with high cellular density. Lytic deferment requires that the phage chromosome be capable of surviving for a limited amount of time in the cell cytoplasm. A number of mechanisms, ranging from circularization to lysogeny, can be implemented to increase chromosome survival. The specific mechanisms used by the different pili/flagellotropic phages to survive lytic deferment remain to be elucidated and are likely diverse, given the independent evolution of this strategy. *In silico* analyses did not detect the presence of plasmid-associated genes in LPEG phages (Pfeifer et al., 2021), but analysis of all analyzed phage genomes with the BACPHLIP algorithm strongly predicted several of the LPEG phages, including ΦCbk-like phages (*p* > 0.95), as temperate (Figure 3; Supplementary Table S10; Supplementary Figure S15). Other LPEG phages, like Brevundimonas phage vB_BsubS-Delta, cluster deep within a large clade of phages shown to confer homoimmunity, but not to integrate into host replicons (Williamson et al., 2001; Lohr et al., 2005; Ceyssens and Lavigne, 2010). This suggests that some LPEG phages may be able to integrate into the host chromosome, while others adopt alternative mechanisms.

This research clearly generates many more questions than it answers, but it may be a first indication that phages infecting the Alphaproteobacteria, and particularly those relying on pili/flagella to infect their hosts, may have co-opted the transcriptional activator that controls the development of these cellular appendages to time their replication in a way that maximizes their chances of finding infectable cells and their dispersal across scattered niches of high bacterial growth. In the absence of phage mutants or homology-predicted functions for the phage genes shown to be CtrA-regulated, the precise molecular mechanisms underpinning lytic deferment cannot be determined and the proposal of such a pathway must remain speculative. However, while other interpretations for the computational and experimental results reported here are possible, the hypothesis of phages leveraging CtrA to implement lytic deferment provides a well-defined evolutionary rationale that is consistent with the recent identification of quorum sensing mechanisms in temperate phages infecting the Bacillales, with the well-established control of Gene Transfer Agents (GTA) by CtrA (Lang et al., 2017), and with the reported presence in different Alphaproteobacteria-infecting phages of homologs for cell-cycle regulators.

## Data availability statement

The datasets and code used in this study can be found in the LPEG_phages repository (https://github.com/ErillLab/LPEG_phages). The *Brevundimonas* phage vB_BsubS-Delta complete genome sequence is available at the NCBI GenBank under accession number MN862068.1.

## Author contributions

PC and IE: conceptualization. EM, SC, and IE: data curation. EM, SA, PC, and IE: formal analysis. PC and RY: funding acquisition. EM, RY, TC, SC, PC, SA, and IE: investigation. EM, PC, and IE: methodology. RY, PC, and IE: project administration. SC, RY, PC, TC, and IE: resources. EM, AFS, and IE: software. JS-S, RY, PC, and IE: supervision. EM and IE: visualization. EM, PC, SA, and IE: writing—original draft. EM, TC, SC, SA, JS-S, AFS, RY, PC, and IE: writing—review and editing.

## Funding

This work was funded in part by the United States National Science Foundation CAREER program award 1552647 to PC, and by grant NIH R35 GM136396 to RY. EM was supported by the UMBC Department of Biological Sciences.

## Conflict of interest

The authors declare that the research was conducted in the absence of any commercial or financial relationships that could be construed as a potential conflict of interest.

## Publisher’s note

All claims expressed in this article are solely those of the authors and do not necessarily represent those of their affiliated organizations, or those of the publisher, the editors and the reviewers. Any product that may be evaluated in this article, or claim that may be made by its manufacturer, is not guaranteed or endorsed by the publisher.

## Footnotes

^1^ https://github.com/ErillLab/LPEG_phages

^2^ https://github.com/ErillLab/ViPhy

^3^ https://github.com/ErillLab/pilitropic_phages
