# Supplementary material for: The transcriptional regulator CtrA controls gene expression in Alphaproteobacteria phages: Evidence for a lytic deferment pathway
Source: Front Microbiol. 2022 Aug 19;13:918015. doi: 10.3389/fmicb.2022.918015 (PMC9437464; doi:10.3389/fmicb.2022.918015)
Supplement: Supplementary file 1 [file Image_1.PDF]

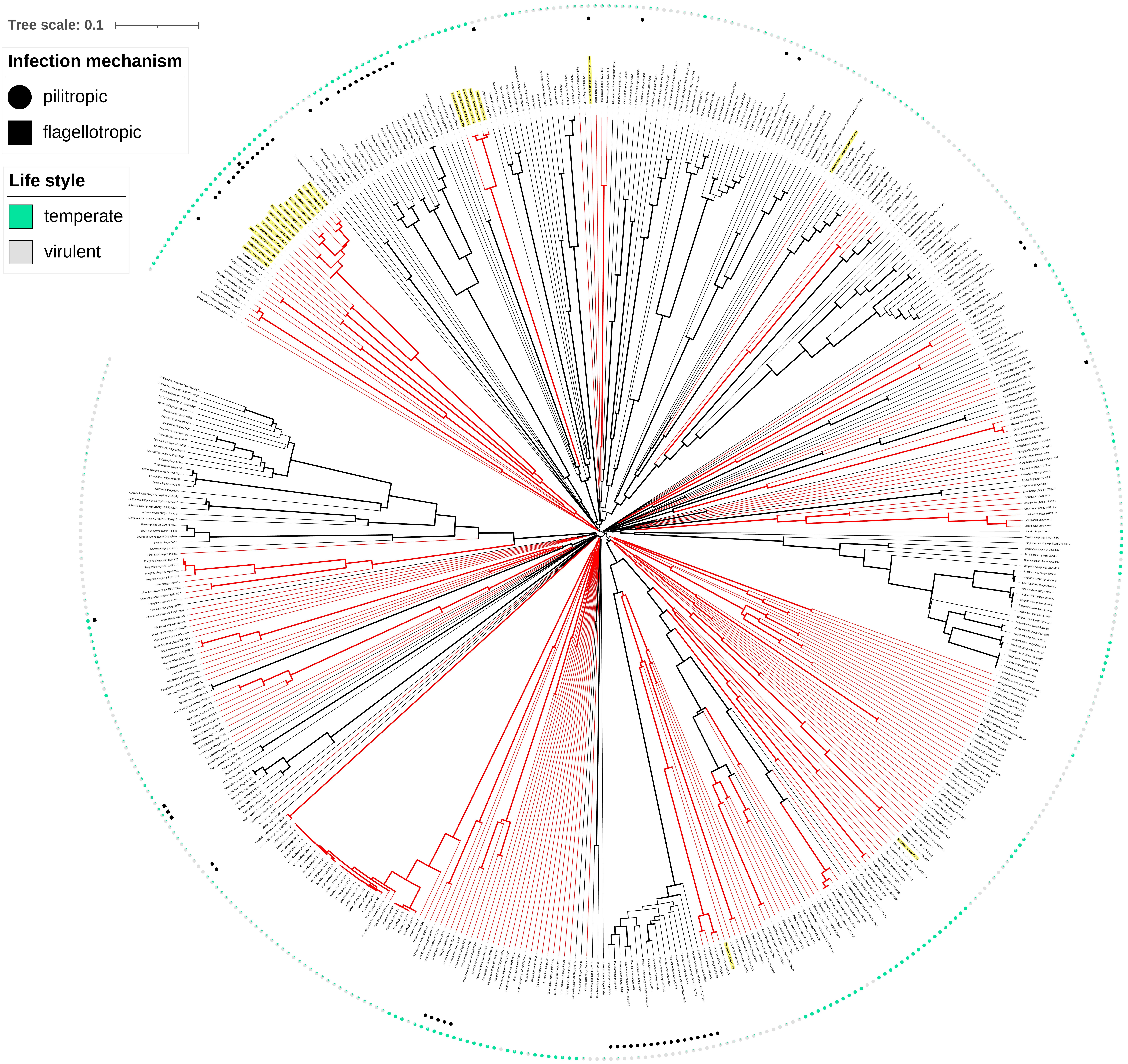

Supplementary Figure 1. Whole-genome phylogeny of vB_BsubS-Delta. Whole-proteome tree of complete bacteriophage genomes. The tree was generated with FASTML using BLAST-derived inter-genomic distances using ViPhy. Midpoint rooting was applied for circular visualization. Bootstrap support values are shown as branch widths, with the thickest branches corresponding to 100% bootstrap support. The branches corresponding to Alphaproteobacteria-infecting phages are colored in red. External filled circles and squares designate, respectively, known pilitropic and flagellotropic phages. Names for LPEG phages are highlighted in yellow. External pie-charts indicate (green) the probability of temperateness assigned by the BACPHLIP algorithm.
