# Supplementary material for: The transcriptional regulator CtrA controls gene expression in Alphaproteobacteria phages: Evidence for a lytic deferment pathway
Source: Front Microbiol. 2022 Aug 19;13:918015. doi: 10.3389/fmicb.2022.918015 (PMC9437464; doi:10.3389/fmicb.2022.918015)
Supplement: Supplementary file 2 [file Image_2.PDF]

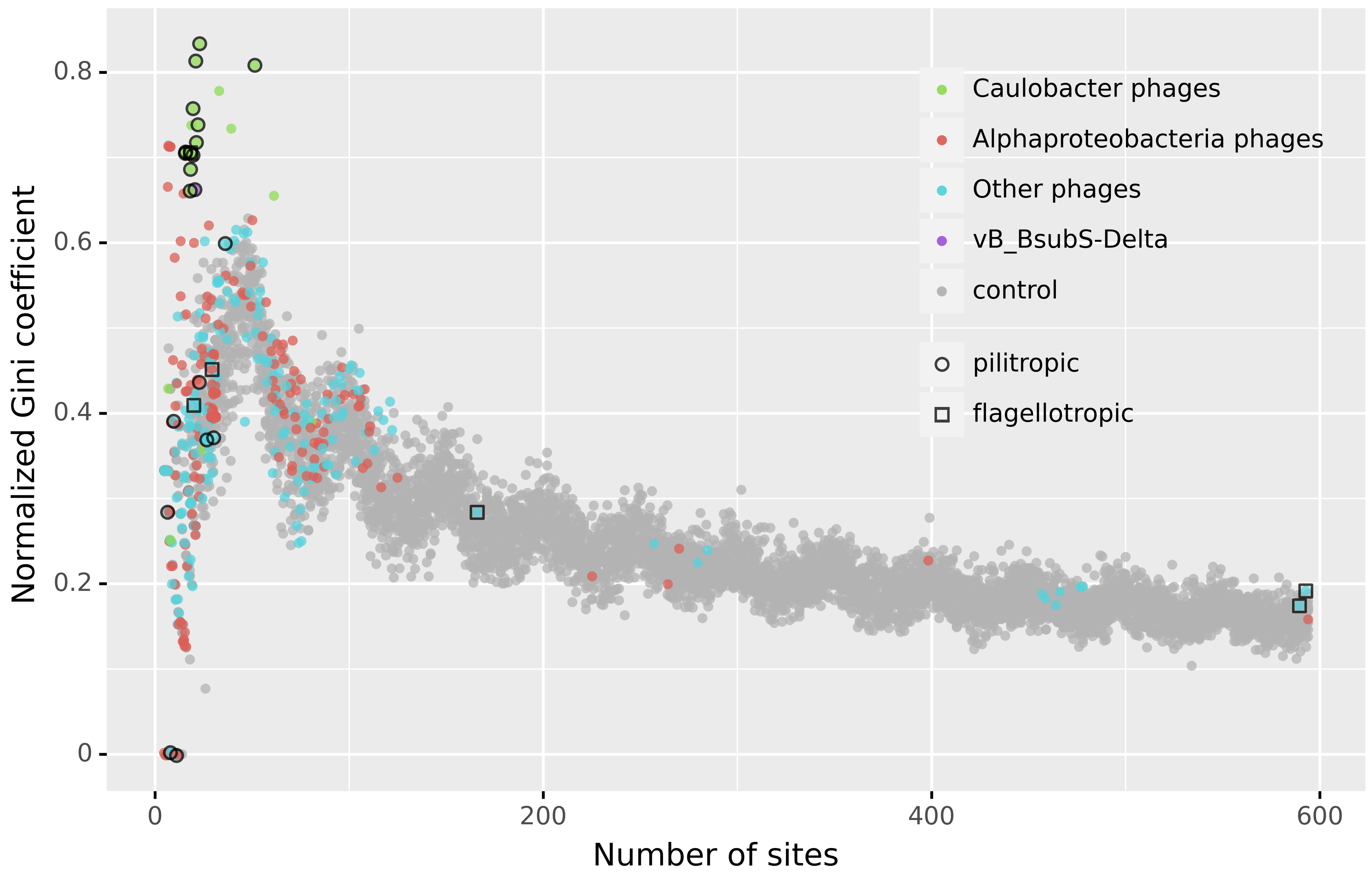

Supplementary Figure 2. Normalized Gini Coefficient vs. number of identified CtrA-binding sites. Each filled circle indicates the normalized Gini coefficient of the genome as a function of the number of putative binding sites detected in its genome sequence. Uniform jitter was applied for clarity. Color coding in filled circles denotes, respectively, *Caulobacter* phages (green), other Alphaproteobacteria phages (red), vB_BsubS-Delta (purple), all the other analyzed phages (cyan) and the negative control (gray). The negative control was obtained by generating 10 random positional distributions for each possible number of sites and computing their normalized Gini coefficient (for a total of 5890 data points). Open circles designate known pilitropic phages, and open squares denote known flagellotropic phages.
