# Supplementary material for: The transcriptional regulator CtrA controls gene expression in Alphaproteobacteria phages: Evidence for a lytic deferment pathway
Source: Front Microbiol. 2022 Aug 19;13:918015. doi: 10.3389/fmicb.2022.918015 (PMC9437464; doi:10.3389/fmicb.2022.918015)
Supplement: Supplementary file 3 [file Image_3.PDF]

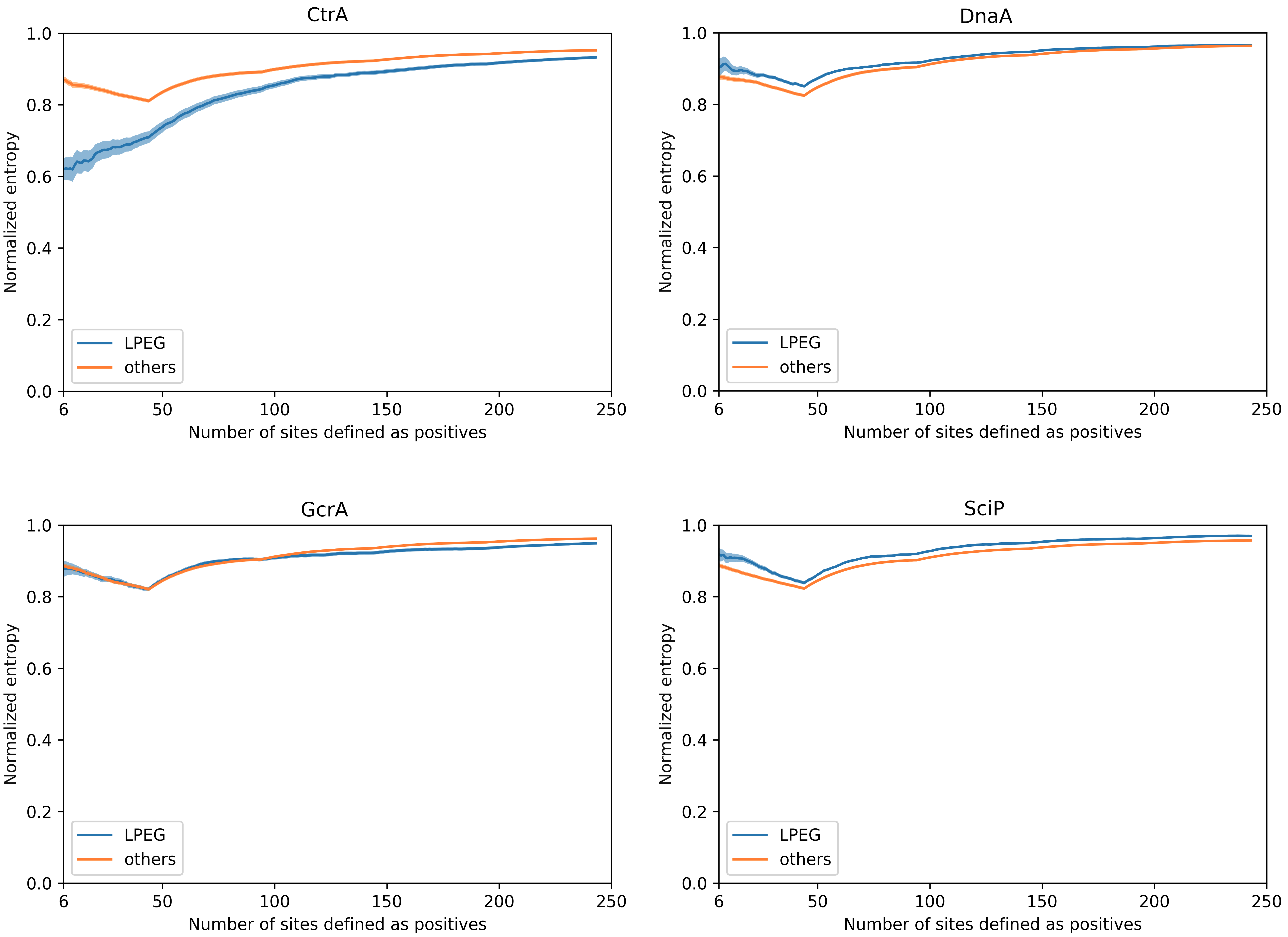

Supplementary Figure 3. Normalized positional entropy curves. For each TF, decreasing PSSM threshold values were used to define different numbers of predicted binding sites (from 6 to 250). For each threshold value, the average normalized positional entropy was calculated for the LPEG set and for all the other phage genomes. The shaded area reports the standard error of the mean. The theoretical expectation is that normalized positional entropy will converge to 1 as the threshold decreases.
