# Supplementary material for: The transcriptional regulator CtrA controls gene expression in Alphaproteobacteria phages: Evidence for a lytic deferment pathway
Source: Front Microbiol. 2022 Aug 19;13:918015. doi: 10.3389/fmicb.2022.918015 (PMC9437464; doi:10.3389/fmicb.2022.918015)
Supplement: Supplementary file 4 [file Image_4.PDF]

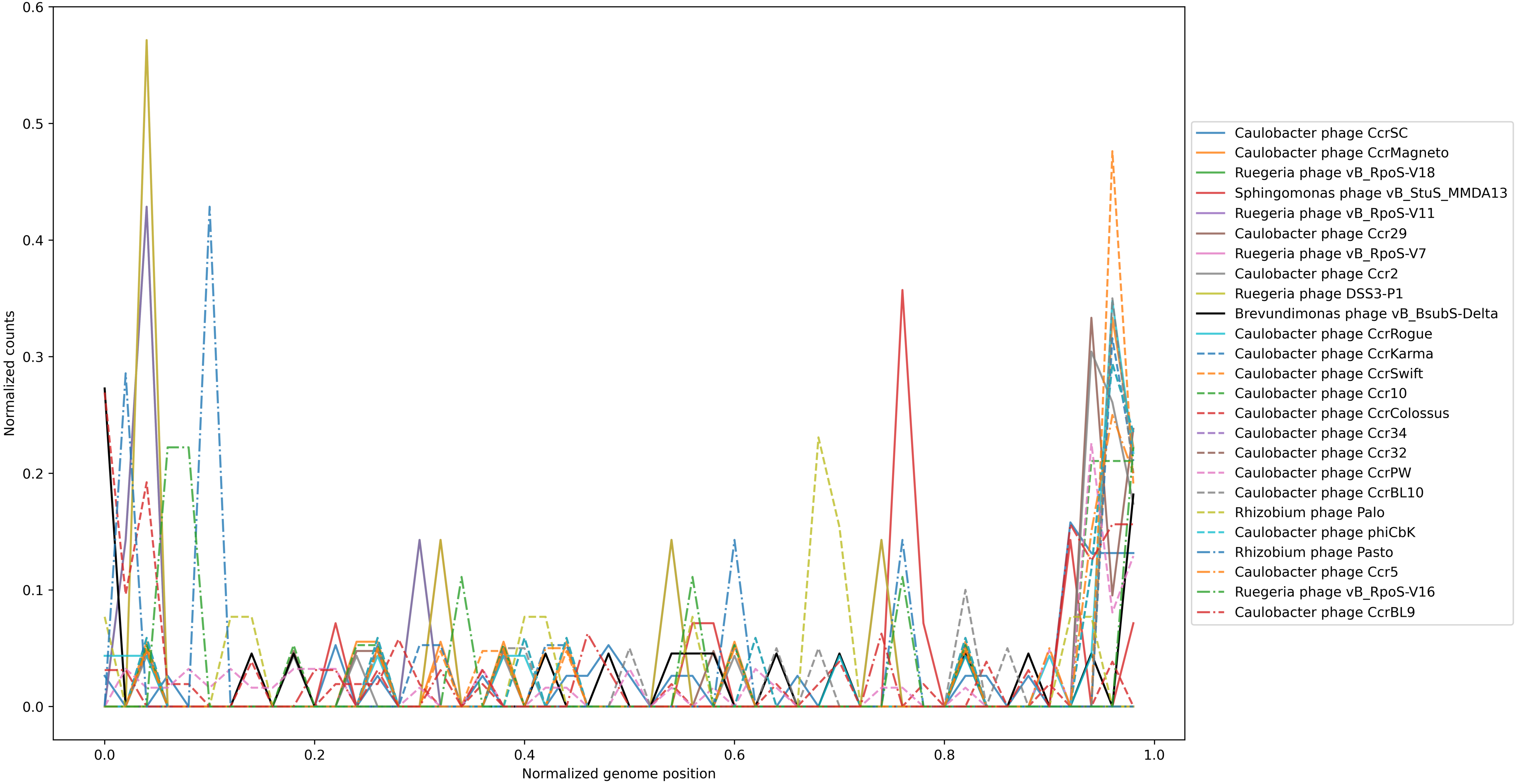

Supplementary Figure 4. Positional distribution of putative CtrA-binding sites in LPEG. Each genome was partitioned into 50 bins. The line plot shows the frequency of putative CtrA-binding sites lying within each bin for each LPEG.
