# Supplementary material for: The transcriptional regulator CtrA controls gene expression in Alphaproteobacteria phages: Evidence for a lytic deferment pathway
Source: Front Microbiol. 2022 Aug 19;13:918015. doi: 10.3389/fmicb.2022.918015 (PMC9437464; doi:10.3389/fmicb.2022.918015)
Supplement: Supplementary file 5 [file Image_5.PDF]

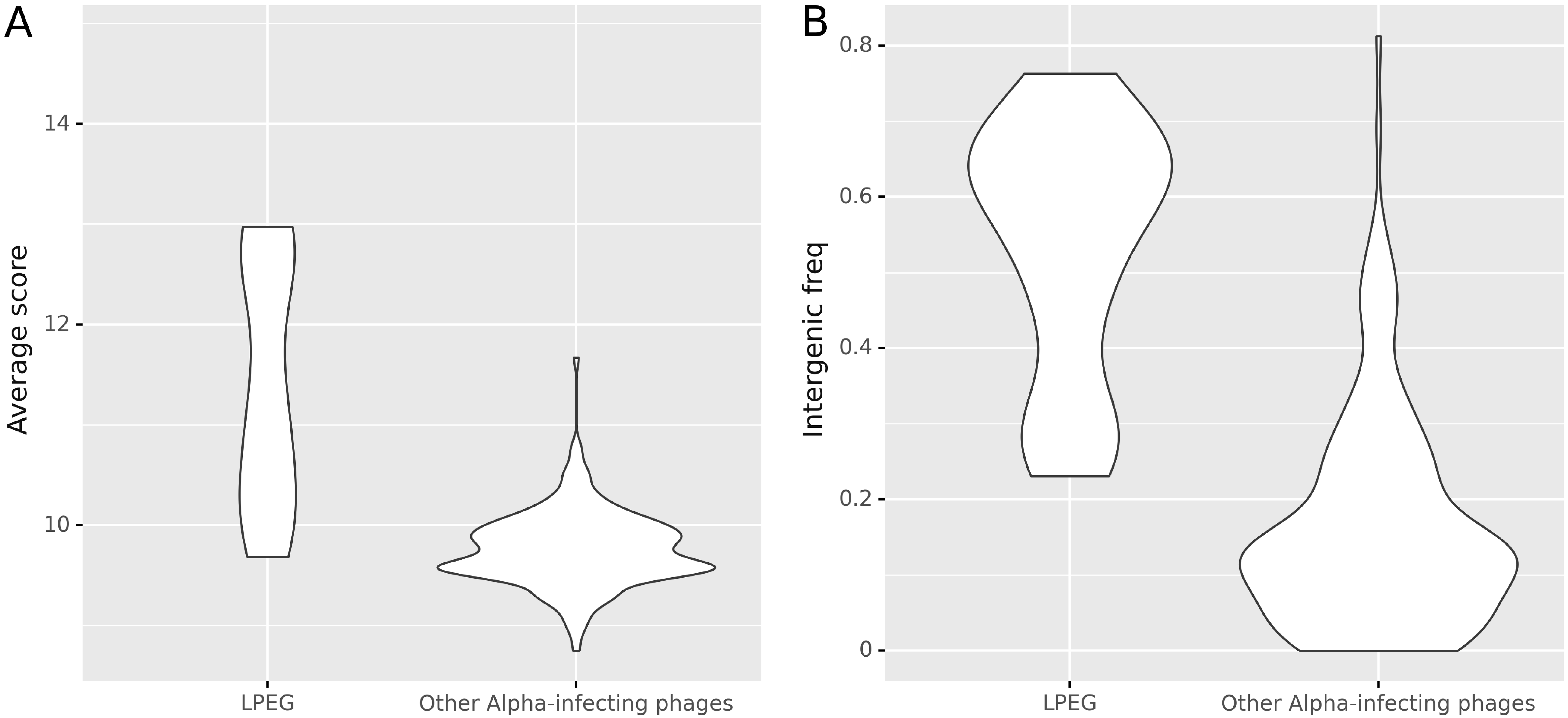

Supplementary Figure 5. Comparison of predicted CtrA-binding sites between LPEG and all the other Alphaproteobacteria-infecting phages. (A) Comparison of the distribution of the average PSSM score for predicted CtrA-binding sites over the entire genome, in LPEG phages and in all the other Alphaproteobacteria-infecting phage genomes. (B) Comparison of the distribution of intergenic frequency for predicted CtrA-binding sites (defined as the proportion of predicted CtrA-binding sites identified in non-coding regions) in LPEG and all the other alphaproteobacteria-infecting phage genomes.
