# Supplementary material for: The transcriptional regulator CtrA controls gene expression in Alphaproteobacteria phages: Evidence for a lytic deferment pathway
Source: Front Microbiol. 2022 Aug 19;13:918015. doi: 10.3389/fmicb.2022.918015 (PMC9437464; doi:10.3389/fmicb.2022.918015)
Supplement: Supplementary file 6 [file Image_6.PDF]

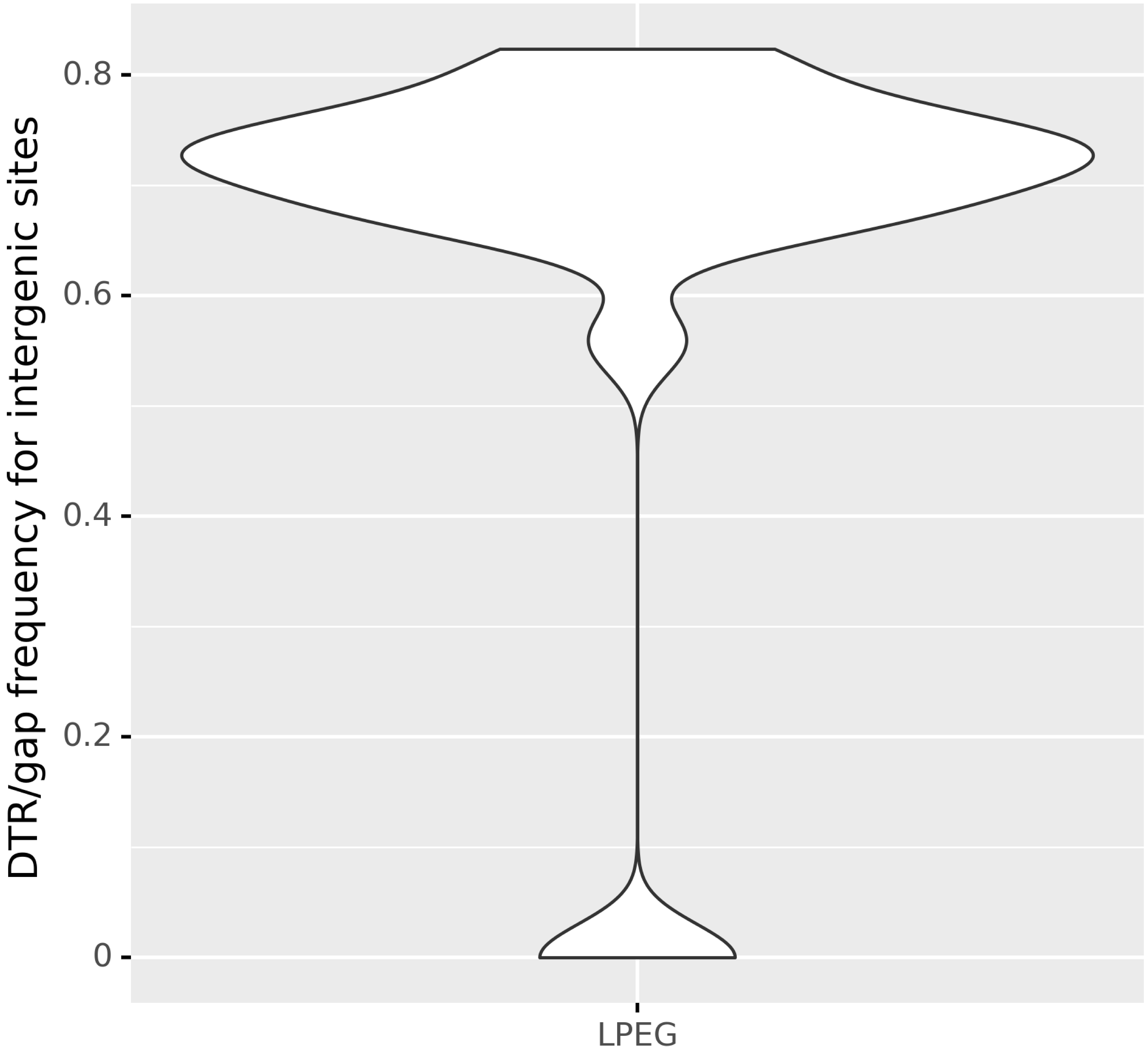

Supplementary Figure 6. Proportion of intergenic CtrA-binding sites mapping to DTR/gap regions in LPEG phages. The plot shows the proportion of intergenic CtrA-binding sites that lie in DTR/gap regions, among all predicted intergenic CtrA-binding sites within each LPEG sequence.
