# Supplementary material for: The transcriptional regulator CtrA controls gene expression in Alphaproteobacteria phages: Evidence for a lytic deferment pathway
Source: Front Microbiol. 2022 Aug 19;13:918015. doi: 10.3389/fmicb.2022.918015 (PMC9437464; doi:10.3389/fmicb.2022.918015)
Supplement: Supplementary file 8 [file Image_8.PDF]

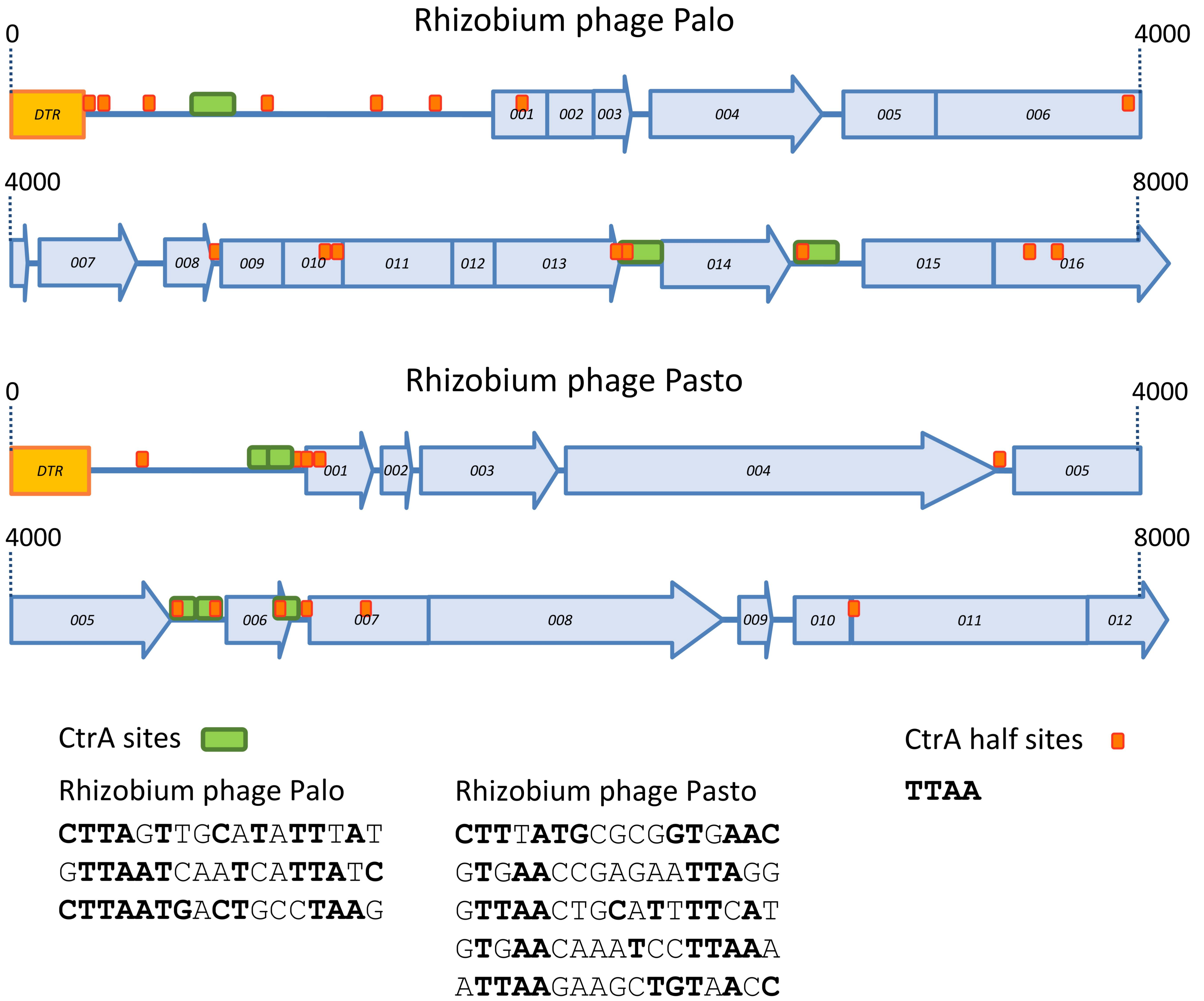

Supplementary Figure 8. High density CtrA-binding site region in *Rhizobium* Pasto and Palo genomes. The positions of putative CtrA-binding sites and half-sites in the first 8,000 bp of the genomes of *Rhizobium* phage Palo and *Rhizobium* phage Pasto are displayed as green and orange boxes, respectively. Their sequences are reported, with consensus-matching bases in bold. DTRs are designated as yellow boxes. The location of protein coding genes is also shown.
