# Supplementary material for: The transcriptional regulator CtrA controls gene expression in Alphaproteobacteria phages: Evidence for a lytic deferment pathway
Source: Front Microbiol. 2022 Aug 19;13:918015. doi: 10.3389/fmicb.2022.918015 (PMC9437464; doi:10.3389/fmicb.2022.918015)
Supplement: Supplementary file 9 [file Image_9.pdf]

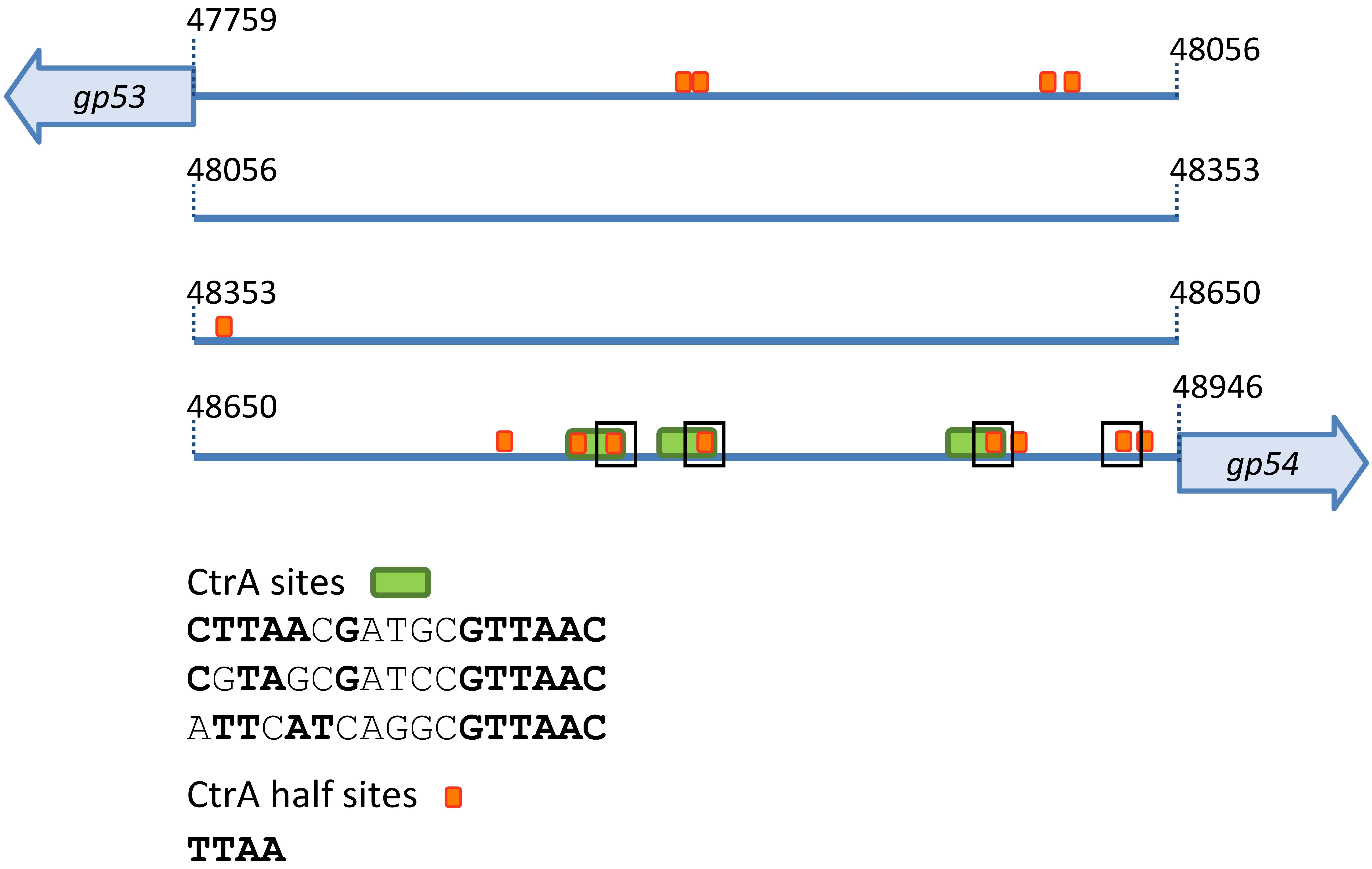

Supplementary Figure 9. *Sphingomonas* phage vB_StuS_MMDA13 gap region diagram. The positions of putative CtrA-binding sites and half-sites in *Sphingomonas phage vB_StuS_MMDA13* gap region are displayed as green and orange boxes, respectively. Their sequences are reported, with consensus-matching bases in bold. The location of protein coding genes is also shown. The direct repeats reported in PMID:32824138 are shown as black open boxes.
