# Supplementary material for: The transcriptional regulator CtrA controls gene expression in Alphaproteobacteria phages: Evidence for a lytic deferment pathway
Source: Front Microbiol. 2022 Aug 19;13:918015. doi: 10.3389/fmicb.2022.918015 (PMC9437464; doi:10.3389/fmicb.2022.918015)
Supplement: Supplementary file 10 [file Image_10.PDF]

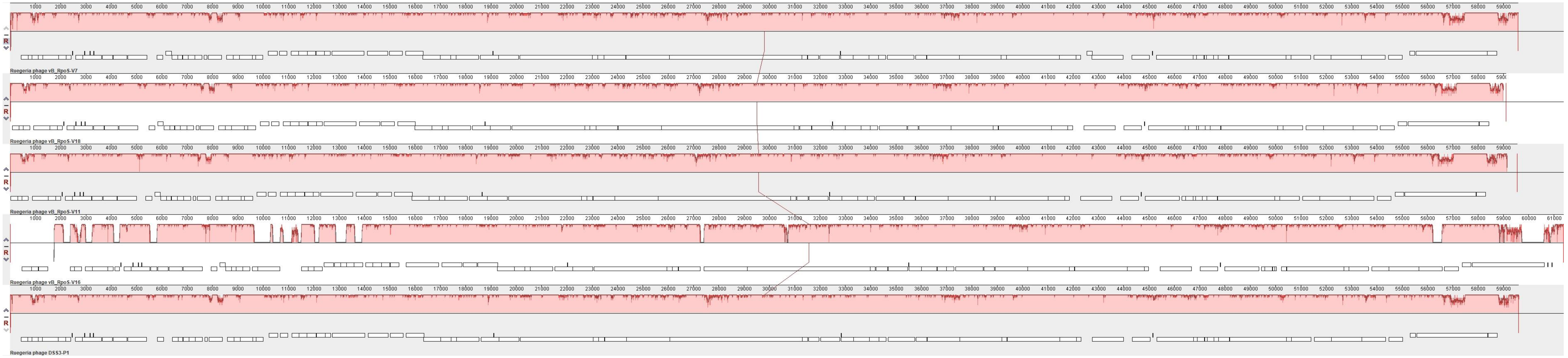

Supplementary Figure 10. Mauve alignment of *Ruegeria* LPEG phage genome sequences. Regions with significant sequence conservation are shown in colors, using the Mauve backbone color scheme. Predicted CtrA-binding sites are shown as blue boxes. Annotated genes are shown as white boxes.
