# Supplementary material for: The transcriptional regulator CtrA controls gene expression in Alphaproteobacteria phages: Evidence for a lytic deferment pathway
Source: Front Microbiol. 2022 Aug 19;13:918015. doi: 10.3389/fmicb.2022.918015 (PMC9437464; doi:10.3389/fmicb.2022.918015)
Supplement: Supplementary file 11 [file Image_11.PDF]

```
                                                                                 60
                                                                                  |
Rp CzcR   MRVLLIEDES EMANLIEITL ASEGIVCDKA SVGVEGLRLG KVGIYDLVIL DLMLPDINGF
Rc CtrA   MRILLVEDDP TTSRSIELML THANLNVYCT DLGEEGIDLA KLYDYDLIPL DLNLPDMNGL
St CtrA   MRVLLIEDEP TTAKAIELML TTEGFNVYTT DLGEEGLDLA KLYDYDIILL DLNLPDMHGY
Sm CtrA   MRVLLIEDDS ATAQSIELML KSESFNVYTT DLGEEGVDLG KLYDYDIILL DLNLPDMSGY
Ml CtrA   MRVLLIEDDS ATAQSIELML KSESFNVYTT DLGEEGVDLG KLYDYDIILL DLNLPDMSGY
Ba CtrA   MRVLLIEDDS AIAQSIELML KSESFNVYTT DLGEEGIDLG KLYDYDIILL DLNLPDMSGY
Cc CtrA   MRVLLIEDDS ATAQTIELML KSEGFNVYTT DLGEEGVDLG KIYDYDLILL DLNLPDMSGI
Bs CtrA   MRVLLIEDDH ATAQSIELML KSEGFNVYTT DLGEEGIDLG KIYDYDMIML DLNLPDMSGL
          **:**:**:     :. **: *     .:     :  .:* **: *. *:  **:: * ** ***: *

                                                                                120
                                                                                  |
Rp CzcR   EILLRLRAAK IKTPILILSS LTDTDQKITS FSSGADDYLT KPFVREELIA RIKAIVRRSK
Rc CtrA   EVLRQLRLAR VDTPILILTG ADDTENKIKG FGFGADDYMT KPFHREELVA RIHAIIRRSK
St CtrA   DVLKKVRVAR VQTPVLILSG INEMDSKVRS FGFGADDYVT KPFHREELVA RIHAVVRRSK
Sm CtrA   EVLRTLRLSK VKTPILILSG MAGIEDKVRG LGFGADDYMT KPFHKDELVA RIHAIVRRSK
Ml CtrA   EVLRTLRLSK VKTPILILSG MAGIEDKVRG LGFGADDYMT KPFHKDELVA RIHAIVRRSK
Ba CtrA   EVLRTLRLSK VKTPILILSG MAGIEDKVRG LGFGADDYMT KPFHKDELIA RIHAIVRRSK
Cc CtrA   DVLRTLRVAK VNTPIMILSG TAEIDTKVKT FAGGADDYMT KPFHKDEMIA RIHAVVRRSK
Bs CtrA   EVLRQLRVGK INTPVMILSG STEIETKVKT FGGGADDYMT KPFHKDELIA RTHAVVRRSK
          ::*  :* .: :.**::**:.       : *:    :. *****:* *** ::*::* * :*::****

                β1     β2         β3     β4          α1            β5     α2     180
                                                                                  |
Rp CzcR   GHAASIFRFD KISVNLDTRS VEVDGKKLHL TNKEYSILEL LILRRGTILT KEMFLNHLYS
Rc CtrA   GHSQSIIRTG KISVNLDAKT VEVGGKPVHL TGKEYQMLEL LSLRKGTTLT KEMFLNHLYG
St CtrA   GHSQSVIRTG KLAVNLDAKT VEVDGARVHL TGKEYAMLEL LSLRKGTTLT KEMFLNHLYG
Sm CtrA   GHAQSVISTG ELIVNLDAKT VEVGGQRVHL TGKEYQMLEL LSLRKGTTLT KEMFLNHLYG
Ml CtrA   GHAQSVISTG ELIVNLDAKT VEVGGQRVHL TGKEYQMLEL LSLRKGTTLT KEMFLNHLYG
Ba CtrA   GHAQSVITTG DLVVNLDAKT VEVAGQRVHL TGKEYQMLEL LSLRKGTTLT KEMFLNHLYG
Cc CtrA   GHAQSVIKTG DIIVNLDAKT VEVNGNRVHL TGKEYQMLEL LSLRKGTTLT KEMFLNHLYG
Bs CtrA   GHAQAIIHTG EIAVNLDGKT VEVHGHRVHL TGKEYQMLEL LSLRKGTTLT KEMFLNHLYG
          **: :::    .  .: **** :: *** *   :** *.*** :*** * **:** ** *********.

                          α3                       β6        β7          237
                                                                          |
Rp CzcR   TVDEPEMKII DVFICKLRKK LSDAAGGRDY IDTVWGRGYM LKEYDELQQK ELLAQGA
Rc CtrA   GMDEPELKII DVFICKLRKK LAEVTGGENY IETVWGRGYV LRDPDQGDLD RRMVVGA
St CtrA   GMDEPELKII DVFICKLRKK LSMACDGENY IETVWGRGYV LRDAEEVTAP VAEVA--
Sm CtrA   GMDEPELKII DVFICKLRKK LANAAGGANY IETVWGRGYV LREPEGSDYL ETA----
Ml CtrA   GMDEPELKII DVFICKLRKK LANAAGGANY IETVWGRGYV LREPEGSDYL ETA----
Ba CtrA   GMDEPELKII DVFICKLRKK LDAVSGNQSY IETVWGRGYV LREPDAEMRE SA-----
Cc CtrA   GMDEPELKII DVFICKLRKK LAASAQGKHH IETVWGRGYV LRDPNEQVSA A------
Bs CtrA   GMDEPELKII DVFICKLRKK LATAAGGKHY IETVWGRGYV LRDPSESTMP SPVSAAA
           :****:*** ********** *      .  : *:*******: *:: .
```

Supplementary Figure 11. Multiple sequence alignment of CtrA sequences. CLULSTALW multiple sequence alignment of representative Alphaproteobacteria CtrA protein sequences. Blue and yellow boxes designate, respectively, the conserved aspartate phosphorylation site and acidic pocket residues. Superimposed legends designate α helices and β sheets mapping to the OmpR C-terminal domain. The CLUSTALW consensus sequence highlights sequence conservation. Sequence abbreviations and accessions are as follows: *S. meliloti* (Sm) (AF288464), *B. abortus* (Ba) (AAC69920), *C. crescentus* (Cc) (KSB90788), *R. capsulatus* (Rc) (WP_055208583), *Rickettsia prowazekii* (Rp) CzcR (CAA14542), *B. subvibrioides* (Bs) (WP_013268628), *Sphingomonas turrisvirgatae* (Rc) (WP_069319698), *Mesorhizobium loti* (Ml) (WP_027062253).
