# Supplementary material for: The transcriptional regulator CtrA controls gene expression in Alphaproteobacteria phages: Evidence for a lytic deferment pathway
Source: Front Microbiol. 2022 Aug 19;13:918015. doi: 10.3389/fmicb.2022.918015 (PMC9437464; doi:10.3389/fmicb.2022.918015)
Supplement: Supplementary file 12 [file Image_12.PDF]

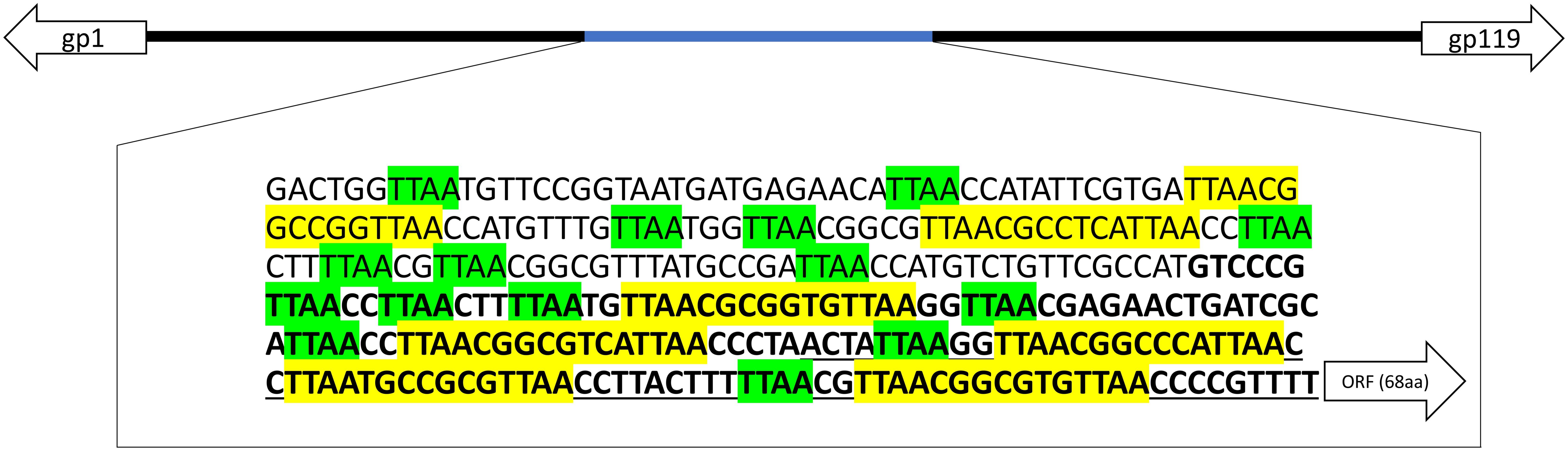

Supplementary Figure 11. Diagram of the *Brevundimonas* phage vB_BsubS-Delta gap region. Detail of the 1.3kb intergenic region between DELTA_1 and DELTA_119 genes. Full CtrA-binding sites are highlighted in yellow; half sites are highlighted in green. A near canonical TTAA-N8-TTAA site is also shown. The DELTA_120 ORF encoding a 68 aa protein is also illustrated with an arrow. Underlined bases were used for the EMSA probe. Bases in boldface were used for beta galactosidase reporter fusions.
