# Supplementary material for: The transcriptional regulator CtrA controls gene expression in Alphaproteobacteria phages: Evidence for a lytic deferment pathway
Source: Front Microbiol. 2022 Aug 19;13:918015. doi: 10.3389/fmicb.2022.918015 (PMC9437464; doi:10.3389/fmicb.2022.918015)
Supplement: Supplementary file 13 [file Image_13.PDF]

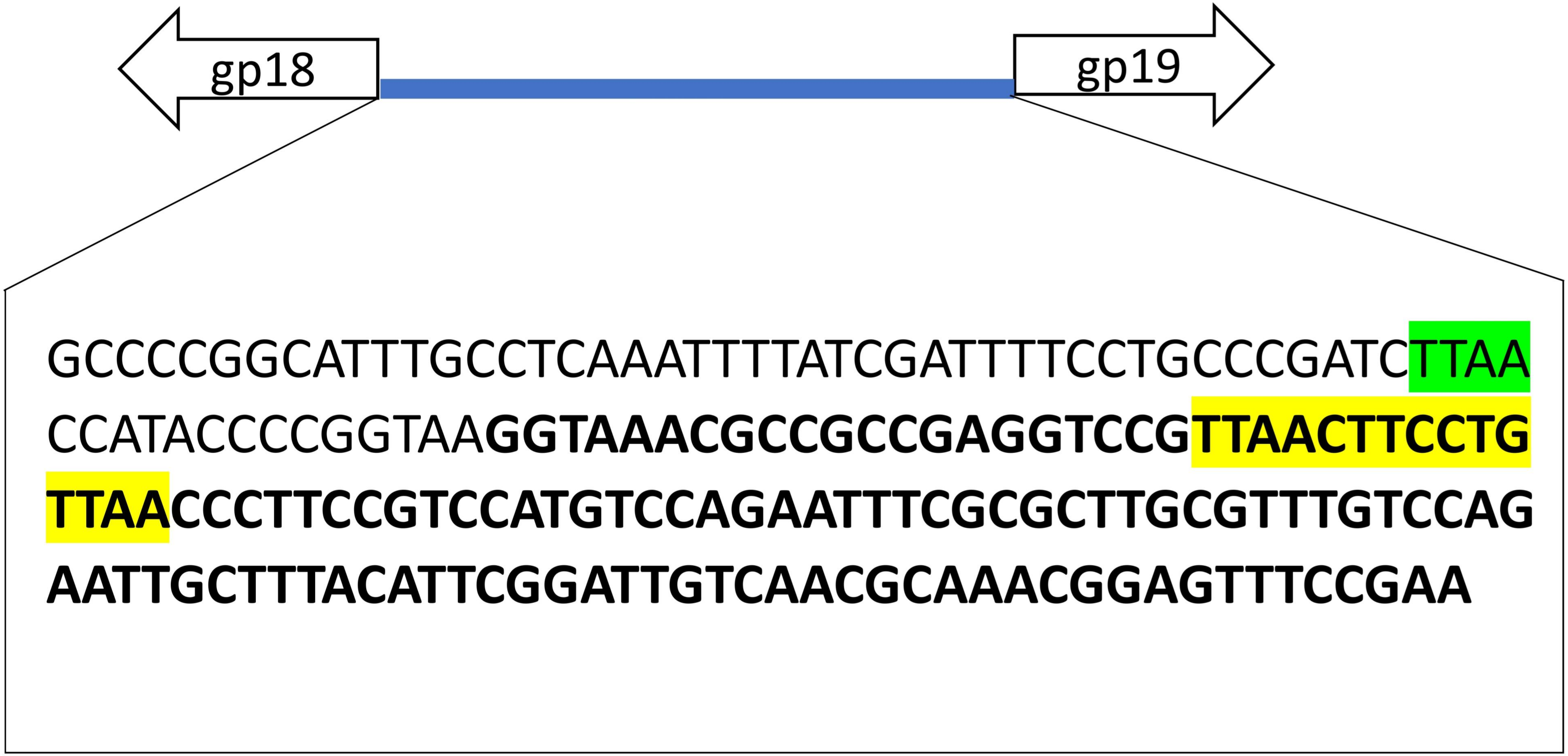

Supplementary Figure 12. Diagram of the *Mesorhizobium* phage Cp1R7A-A1 gap region. Intergenic region between Cp1R7AA1_018 and Cp1R7AA1_019 genes. Full CtrA-binding sites are highlighted in yellow; half sites are highlighted in green. Bases in boldface were used for beta galactosidase reporter fusions.
