# Supplementary figures and images for: The transcriptional regulator CtrA controls gene expression in Alphaproteobacteria phages: Evidence for a lytic deferment pathway
Source: Front Microbiol. 2022 Aug 19;13:918015. doi: 10.3389/fmicb.2022.918015 (PMC9437464; doi:10.3389/fmicb.2022.918015)

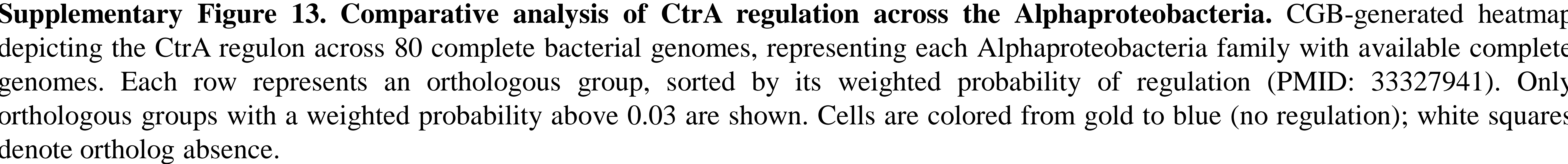

Supplement: Supplementary file 14 [file Image_14.PDF]
