# Supplementary material for: The transcriptional regulator CtrA controls gene expression in Alphaproteobacteria phages: Evidence for a lytic deferment pathway
Source: Front Microbiol. 2022 Aug 19;13:918015. doi: 10.3389/fmicb.2022.918015 (PMC9437464; doi:10.3389/fmicb.2022.918015)
Supplement: Supplementary file 15 [file Image_15.PDF]

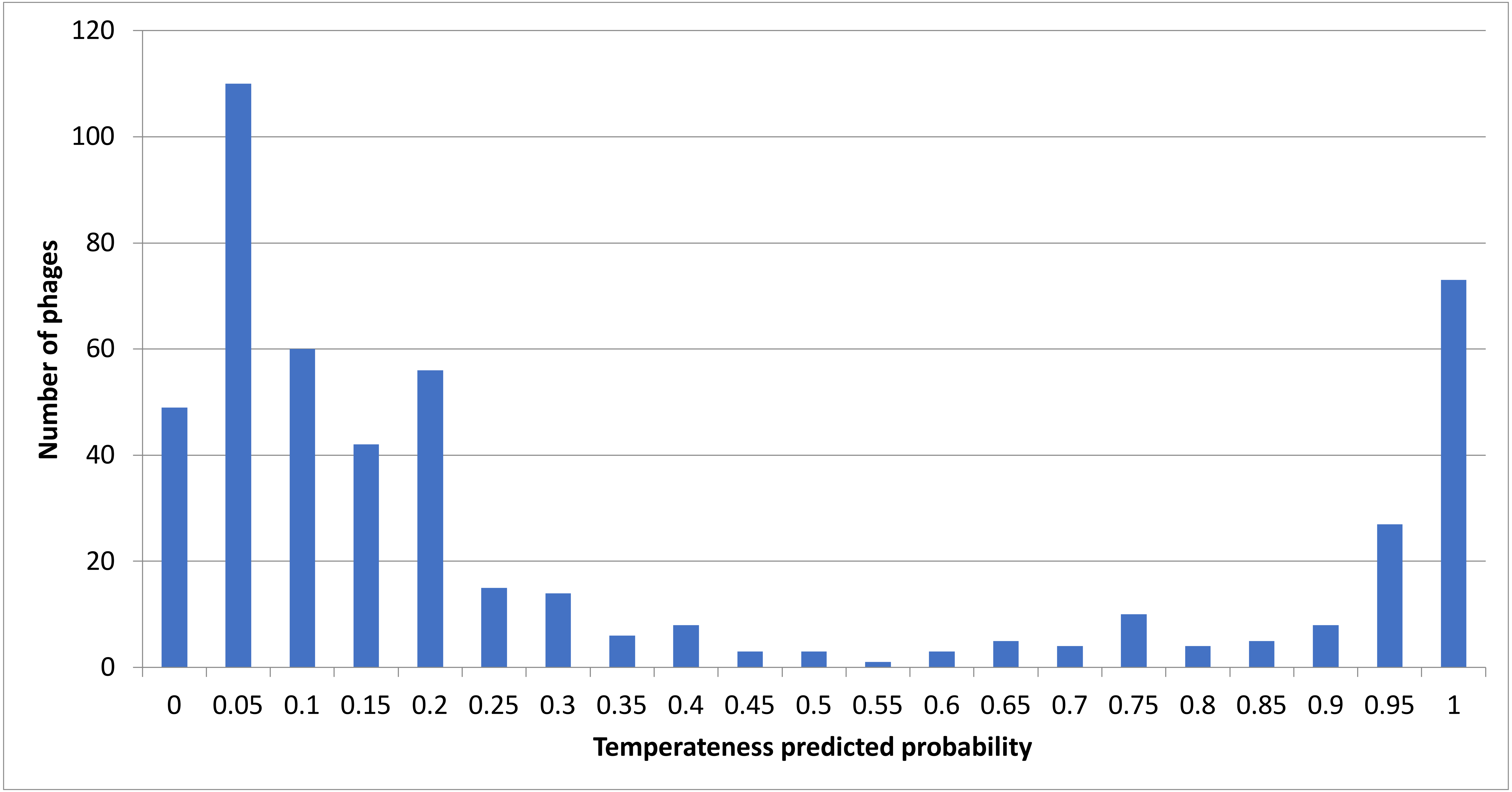

Supplementary Figure 14. BACPHLIP prediction distribution. Distribution of predicted temperate probabilities for the entire set of analyzed phages.
