# Supplementary material for: The transcriptional regulator CtrA controls gene expression in Alphaproteobacteria phages: Evidence for a lytic deferment pathway
Source: Front Microbiol. 2022 Aug 19;13:918015. doi: 10.3389/fmicb.2022.918015 (PMC9437464; doi:10.3389/fmicb.2022.918015)
Supplement: Supplementary file 16 [file Table_1.PDF]

Supplementary Table 1. List of strains, plasmids and primers used in this study.

Strains	Description	Source
E. coli		
Alpha-select	F- *deo*R *end*A1 *rec*A1 *rel*A1 *gyr*A96 *hsd*R17(r_k^-, m_k^+) *sup*E44 *thi*-1 *pho*A Δ(*lac*ZYA-*arg*F)U169 Φ80*lac*ZΔM15 λ^-	Bioline
PC0312	Alpha-select; pAR0001	
PC0341	Alpha-select; pSA1002	This study
PC0336	Alpha-select; pSA1003	This study
PC0335	Alpha-select; pSA1004	This study
PC0345	Alpha-select; pSA1005	This study
C. crescentus		
NA1000	Wild-type	
LS2195	a temperature sensitive allele of *ctrA*	PMID: 8548829
PC0343	NA1000; pSA1002	This study
PC0344	LS2195; pSA1002	This study
PC0339	NA1000; pSA1003	This study
PC0340	LS2195; pSA1003	This study
PC0337	NA1000; pSA1004	This study
PC0338	LS2195; pSA1004	This study
PC0346	NA1000; pSA1005	This study
PC0347	LS2195; pSA1005	This study

Plasmids	Description	Source
plac290	low copy number reporter plasmid	PMID: 1392079
pET28a	Plasmid for creating N-ter 6X histag for expression	Novagen
pAR0001	*ctrA* + pET28a	This study
pSA1002	P*delta_120* + plac290	This study
pSA1003	P*cbk_gp005* + plac290	This study
pSA1004	P*cbk_gp018* + plac290	This study
pSA1005	P*Meso_gp19* + plac290	This study
**Primers**	**Sequence**	**Notes**
ctrApET28aF	GCAAATGGGTCGCGGATCCGAATTCATGCGCGTACTGTTGATCGAG	
ctrApET28aR	GGTGCTCGAGTGCGGCCGCAAGCTTCAGGCGGCGTTAACCTGCTC	
delta120betagalF	GCACGAACCCGCTGAATGGGAATTCGTCCCGTTAACCTTAACTTTTAATG	
delta120betagalR	ATAGCTGTTTCCTGTGTGAAAGCTTAAAACGGGGTTAACACGC	
phicbkgp5promoterF	GCACGAACCCGCTGAATGGGCCGCCTTCGCCCCCTGGCG	
phicbkgp5promoterR	ATAGCTGTTTCCTGTGTGAAGGCGGTTTTCTCCGAAGGCG	
cbkgp18plac290F	GCACGAACCCGCTGAATGGGGTCATCATTCCTACCGTCTA	
cbkgp18plac290R	ATAGCTGTTTCCTGTGTGAATTTGGAAGCTCCGAAGGAAG	
Mesopromoter019F	GCACGAACCCGCTGAATGGGGGTAAACGCCGCCGAGGTC	
Mesopromoter019R	ATAGCTGTTTCCTGTGTGAATTCGGAAACTCCGTTTGCGT	
EMSADELTA_120F	ACTATTAAGGTTAACGGCCCATTAACCTTAATGCCGCGTTAACCTTACTTTTTAACGTTAACGGCGTGTTAACCCCGTTTT	EMSA DNA probe
EMSADELTA_120R	AAAACGGGGTTAACACGCCGTTAACGTTAAAAAGTAAGGTTAACGCGGCATTAAGGTTAATGGGCCGTTAACCTTAATAGT	EMSA DNA Probe
EMSAcbkgp5WTF	CCGCCTTCGCCCCCTGGCGCCGCGTTAACGGTTTGTTAACATATCGGCGCCAGGGTCGCCCTAGCCCGGCGCGTCGCCGCG	EMSA DNA

	CCTTCGGAGAAAACCGCC	probe
EMSAcbkgp5WTR	GGCGGTTTTCTCCGAAGGCGCGGCGACGCGCCGGGCTAGGGCGACCCTGGCGCCGATATGTTAACAAACCGTTAACGCGGCGCCAGGGGGCGAAGGCGG	EMSA DNA probe
EMSAcbkgp5mutF	CCGCCTTCGCCCCCTGGCGCCGCGGGCCCGGTTTGGGCCCATATCGGCGCCAGGGTCGCCCTAGCCCGGCGCGTCGCCGCGCCTTCGGAGAAAACCGCC	EMSA DNA probe
EMSAcbkgp5mutR	GGCGGTTTTCTCCGAAGGCGCGGCGACGCGCCGGGCTAGGGCGACCCTGGCGCCGATATGGGCCCAAACCGGGCCCGCGGCGCCAGGGGGCGAAGGCGG	EMSA DNA probe
EMSAcbkgp18WTF	GCTCTTGCTGGCCTGTTTGTGGCGCTTTTAACGGTTTGTTAACCCCTGGCCCTCTAGGGTCGAATTCCCGGCGCGGCAATCCCGCCGCCCCGGCCTTCCTTCGGAGCTTCCAAA	EMSA DNA probe
EMSAcbkgp18WTR	TTTGGAAGCTCCGAAGGAAGGCCGGGGCGGCGGGATTGCCGCGCCGGGAATTCGACCCTAGAGGGCCAGGGGTTAACAAACCGTTAAAAGCGCCACAAACAGGCCAGCAAGAGC	EMSA DNA probe
EMSAcbkgp18mutF	GCTCTTGCTGGCCTGTTTGTGGCGCTTGGCCCGGTTTGGGCCCCCCTGGCCCTCTAGGGTCGAATTCCCGGCGCGGCAATCCCGCCGCCCCGGCCTTCCTTCGGAGCTTCCAAA	EMSA DNA probe
EMSAcbkgp18mutR	TTTGGAAGCTCCGAAGGAAGGCCGGGGCGGCGGGATTGCCGCGCCGGGAATTCGACCCTAGAGGGCCAGGGGGGCCCAAACCGGGCCAAGCGCCACAAACAGGCCAGCAAGAGC	EMSA DNA probe
EMSAmesogp19WTF	GGTAAACGCCGCCGAGGTCCGTTAACTTCCTGTTAACCCTTCCGTCCATGTCCAGAATTTCGCGCTTGCGTTTGTCCAGAATTGCTTTACATTCGGATTGTCAACGCAAACGGAGTTTCCGAA	EMSA DNA probe
EMSAmesogp19WTR	TTCGGAAACTCCGTTTGCGTTGACAATCCGAATGTAAAGCAATTCTGGACAAACGCAAGCGCGAAATTCTGGACATGGACGGAAGGGTTAACAGGAAGTTAACGGACCTCGGCGGCGTTTACC	EMSA DNA probe
EMSAmesogp19mutF	GGTAAACGCCGCCGAGGTCCGGGCCCTTCCTGGGCCCCCTTCCGTCCATGTCCAGAATTTCGCGCTTGCGTTTGTCCAGAATTGCTTTACATTCGGATTGTCAACGCAAACGGAGTTTCCGAA	EMSA DNA probe
EMSAmesogp19mutR	TTCGGAAACTCCGTTTGCGTTGACAATCCGAATGTAAAGCAATTCTGGACAAACGCAAGCGCGAAATTCTGGACATGGACGGAAGGGGGCCCAGGAAGGGCCCGGACCTCGGCGGCGTTTACC	EMSA DNA probe
